# Supplementary material for: Factors that influence market participation among traditional beef cattle farmers in the Meatu District of Simiyu Region, Tanzania
Source: PLoS One. 2021 Apr 1;16(4):e0248576. doi: 10.1371/journal.pone.0248576 (PMC8016299; doi:10.1371/journal.pone.0248576)
Supplement: S1 Questionnaire — (PDF) [file pone.0248576.s001.pdf]

S1. Questionnaire in English

A questionnaire on the Factors that Influence Market Participation among Traditional Beef Cattle Farmers in the Meatu District of Simiyu Region, Tanzania.

Dear respondents.

I am Cornel A. Kibona, a PhD student (registration No. 49170080) at Jilin Agricultural University-China, pursuing a doctoral degree (PhD) in Agricultural Economics and Management. I am conducting a research on the topic "*Analysis of the Influencing Factors of Traditional Beef Cattle Farmers' Market Participation in the Meatu District of Simiyu Region, Tanzania*". I kindly ask for your cooperation and support in responding to the questions given on the questionnaire. The information obtained will be used only for academic purposes and handled confidentially. No any personal information will be disclosed for public.
Thank you very much for your cooperation.

Best regards
Cornel Anyisile Kibona

Questionnaire on the Influencing Factors of Traditional Beef Cattle Farmers' Market Participation in the Meatu District of Simiyu Region, Tanzania

Region…………**District**……………..**Ward**…………..**Village...........Respondent's Names**…………………**Phone number**…………………..

SECTION A: Background Information

1. Household head's sex 1) male 2) female
2. Age……………..years
3. Marital status 1) single 2) married 3) Divorced 4) widowed
4. Education level………………….years and (Tick where appropriate).
    1=No formal education [ ] 2=Primary education [ ] 3= Secondary education [ ] 4=College education [ ]
5. What is your primary occupation? 1= Wage employment [ ] 2= beef cattle farming [ ] 3= Business [ ] 4= others (specify)
6. What is your secondary occupation?
  1= Wage employment [ ] 2= Business [ ] 3= Crop production [ ] 4= others (specify)
7. How many family members are involved in beef cattle production?

**Number of Adults ≥ 18 years**		**Number of Children ≤ 18 years**	
Male	Female	Male	Female

SECTION B: BEEF CATTLE PRODUCTION INFORMATION

8. What is your current beef cattle herd size...?

9. For what purpose do you keep beef cattle? 1= for prestige [ ] 2= way of life [ ] 3= store of wealth [ ] 4= security/insurance [ ] 5= Food 6= source of income [ ] 7= commercial purpose [ ]

10. For how long have you been keeping beef cattle (years) ………………………………

11. What is your main source of labour used in beef cattle production?

1= Family [ ] 2= Hired [ ] 3= both family and hired [ ]

12. Variable inputs costs for producing each beef cattle (Please fill the table below)

**Variable costs**	**Unit of measurement**	**Frequency (Twice a week or a month)**	**Unit cost**	**Total cost/per year per cattle**
Commercial Minerals				
Labor for herding				
Drugs/medication				
De-worming				
Dipping/Spraying				
Veterinary/breeding services				
Water				
Feeds(grazing)				
Others (Specify)				

13. Do you have access to veterinary services? 1=Yes [ ] 2=No [ ]

14. Do you have access to farm credits 1= Yes [ ] 2= No [ ]

If yes, name the institution from which you access the credits 1= Commercial banks [ ] 2=SACCOs [ ] 3= Microfinance [ ] 4 = others (specify) [ ]

If No, why …………………………………………………..

15. What are the main constraints facing beef cattle production?

1=Drought (shortage of water and pastures) [ ] 2=High costs for buying drugs [ ] 3=Lack of fund [ ] 4=Presence of wild animals [ ] 5=Theft of cattle [ ] 6=Prevalence of diseases [ ] 7=Few cattle dipping facilities in some areas [ ] 10=Lack of credit facilities [ ]

16. What is your current size of your grazing land………………… (Hectares)

17. Which breeds of beef cattle do you own? Indicate the number in each category?

**Category**	**Number**
Exotic	
Crossbreeds	
Local indigenous	

18. What is your current beef herd structure? Fill in the table below

**Category**	**Number**		
	**Local/indigenous**	**Exotic**	**Cross breeds**
Bulls			

Steers/oxen			
Cows			
Heifers			

19. How did you acquire your beef cattle? 1) Inherited 2) purchased at local market 3) Gifts 4) Others (specify) …………

20. What is your major source of income? ....

21. What are your other sources of income? 1) Sales of milk 2) Butter/ghee 3) Hides and skins 4) Sale of crops 5) others (specify) ……………………………………

22. How much income on average from other sources per year.................. Tanzanian shillings

SECTION C: Cattle Sales (Marketing)

23. Have you purchased any beef cattle since January last year? Yes [ ] No [ ]; if yes, how many………

24. Have you sold any beef cattle since January last year? Yes [ ] No [ ]; if yes, complete the complete table

Category	Number sold		Age of cattle sold	Average price	Reason for sale
	Local	Exotic			
Bulls					
Steers/oxen					
Cows					
Heifer					

Reasons for Sale: 1) Pay school fees 2) Pay medical bills 3) Purchase food 4) Animal Sick 5) Purchase household items 6) Animal was old (Culling) 7) Others (specify)…………………

25. Where do you normally sell your cattle? 1= primary auctions market [ ] 2= Abattoirs [ ] 3= Middlemen [ ] 4= Traders [ ] 5.Butchery 6= Secondary auction market. 7=others (specify) [ ]

26. Why do you prefer that market; 1=use of weighing facilities [ ] 2=It is nearby the livestock farmers 3=Good environment for selling [ ] 4=No other alternative market [ ] 6=others…..specify [ ]

27. How do you fix the prices of beef cattle?
1=Take market prices [ ] 2=calculate cost involved [ ] 3=other (specify)…… [ ]

28. How do you determine the price per cattle?
1) Live weight 2) Age 3) Physical appearance 4) Others (specify)…………………………

29. Do you belong to any farmers' cooperatives? Yes [ ] No [ ]; if yes what benefits have you gained from it? 1) Training 2) Study tour 3) Marketing 4) Others (specify) ………………

30. How far are the beef cattle markets from your home? …………….. (Kms)

31. How do you transport your beef cattle to the markets ……………………………………

32. Which months in the previous year did you sell more beef cattle. Please complete the table below

Category	Number of Cattle Sold	Average sold Price

January		
February		
March		
April		
May		
June		
July		
August		
September		
October		
November		
December		

33. Please indicate marketing costs you face when selling beef cattle

**Variable costs**	**Total cost per beef cattle(Tshillings)**
Labor (loading and unloading)	
Transportation cost	
Hidden cost (waiting time, etc.)	
Communication	
Market fees	
Others (specify)	

34. Are you aware of beef cattle prices in other markets? Yes [ ] No [ ] if yes, where do you get the information?

1) Fellow Cattle Keepers 2) Family member 3) NGOs
4) Farmer Association/cooperatives 5) Radio 6) News Papers

35. What are the main beef cattle marketing constraints?

1) Low prices 2) Lack / low numbers of traders 3) Traders default to pay 4) Inaccessibility to cattle market 5) Inaccessibility to market news and information 6) Poor road Condition 7) Quarantine 8) Others (specify……………………

36. Do you practice beef cattle fattening? Yes [ ] No [ ]; if yes, what feeds and cost involved per cattle?.Complete the table below

**Variable costs**	**Unit amount**	**Unit cost**	**Fattening period**	**Age of cattle for fattening**

37. What is the average selling price of fattened beef cattle?.................................Tshs.

THANK YOU FOR YOUR VALUABLE INFORMATION
