# Supplementary material for: Factors that influence market participation among traditional beef cattle farmers in the Meatu District of Simiyu Region, Tanzania
Source: PLoS One. 2021 Apr 1;16(4):e0248576. doi: 10.1371/journal.pone.0248576 (PMC8016299; doi:10.1371/journal.pone.0248576)
Supplement: S2 Questionnaire — (PDF) [file pone.0248576.s002.pdf]

S2. Questionnaire in Swahili

Sababu ambazo zinaathiri Ushiriki wa Soko kati ya Wakulima wa Ng'ombe wa Jadi katika Wilaya ya Meatu Mkoa wa Simiyu, Tanzania.

Wapendwa wahojiwa.

Mimi ni **Cornel A. Kibona**, mwanafunzi wa Ph.D. (usajili No. 49170080) katika Chuo Kikuu cha Kilimo cha Jilin-China, nikisoma digrii ya uzamivu (Ph.D.) katika Uchumi na Usimamizi wa Kilimo. Ninafanya utafiti juu ya mada "Uchambuzi wa Sababu za Ushawishi wa Ushiriki wa Wafugaji wa kienyeji katika Soko la Ng'ombe katika Wilaya ya Meatu Mkoa wa Simiyu, Tanzania ". Ninaomba ushirikiano na msaada wako kujibu maswali yaliyotolewa kwenye dodoso. Habari itakayopatikana itatumika tu kwa madhumuni ya kitaaluma na kushughulikiwa kwa siri. Hapana, habari yoyote ya kibinafsi itafunuliwa kwa umma.
Asante sana kwa ushirikiano wako.

Kila la heri
Cornel Anyisile Kibona

Hojaji juu ya Sababu za Ushawishi wa Ushiriki wa Wafugaji wa kienyeji katika Soko la Ng'ombe katika Wilaya ya Meatu Mkoa wa Simiyu, Tanzania.

Mkoa…………**Wilaya**……………..**Kata**…………………**Kijiji**..**Jina la Muhojiwa**…………………**Namba ya simu**………………………

SEHEMU A: Maelezo ya Asili

1. Jinsia ya kiongozi wa kaya 1) mwanamume 2) mwanamke
2. Umri…………….miaka
3. Hali ya ndoa 1) Hujaolewa 2) Umeolewa 3) Talaka 4) mjane
4. Kiwango cha elimu ………………….miaka (weka alama ya tiki mahali inapofaa).
1 = Hakuna elimu rasmi [] 2 = Elimu ya msingi [] 3 = Elimu ya sekondari [] 4 = Elimu ya chuo na chuo kikuu []
5. Kazi yako ya msingi ni ipi? 1 = Ajira ya mshahara [] 2 = ufugaji wa ng'ombe wa nyama [] 3 = Biashara [] 4 = Vingine (taja)
6. Kazi yako ya ziada ni ipi?
1 = Ajira ya mshahara [] 2 = Biashara [] 3 = Uzalishaji wa mazao [] 4 = vingine (taja)
7. Ni wanafamilia wangapi wanaohusika katika uzalishaji wa ng'ombe wa nyama?

**NIdadi ya watu wazima wenye miaka 18 na zaidi**		**Idadi ya watoto chini ya miaka 18**	
Mwanaume	Mwanamke	Mwanaume	Mwanamke

SEHEMU B: HABARI ZA UZALISHAJI WA NG'OMBE

8. Una ng’ombe kiasi gani kwa sasa...?

9. Je! Unafuga ng'ombe wa nyama kwa kusudi gani? 1 = kwa umaarufu [] 2 = hali ya maisha [] 3 = duka la utajiri [] 4 = usalama / bima [] 5 = Chakula 6 = chanzo cha mapato [] 7 = madhumuni ya kibiashara []

10. Kwa muda gani umekuwa ukifuga ng'ombe wa nyama (miaka)………………………………

11. Je! Chanzo chako kikuu cha wafanyakazi ni kipi katika uzalishaji wa ng'ombe wa nyama?

1 = Familia [] 2 = Kuajiriwa [] 3 = wote familia na walioajiriwa []

12. Variable inputs costs for producing each beef cattle (Please fill the table below)

**Gharama anuwai**	**Kitengo cha kipimo**	**Mzunguko (Mara mbili kwa wiki au mwezi)**	**Gharama ya kitengo**	**Jumla ya gharama / kwa mwaka kwa ng'ombe**
Madini chumvi				
Gharama za wachunga mifugo				
Dawa/matibabu				
Dawa za minyoo				
Gharama za josho kuua kupe				
Huduma za kigan kwa mifugo / ufugaji				
Maji				
Chakula (malisho)				
Vingine (taja)				

13. Je! Unapata huduma ya kigani ya mifugo? 1 = Ndio [] 2 = Hapana []

14. Je! Unapata mikopo kwa ajili ya ufugaji? 1 = Ndio [] 2 = Hapana []

Ikiwa ndio, taja taasisi ambayo unapata mikopo 1 = Benki za kibiashara [] 2 = SACCOs [] 3 = Taasisi ndogo ndogo za kifedha [] 4 = zingine (taja) []. Ikiwa Hapana, kwa nini …………………

15. Je! Ni vizuizi vipi vikuu vinavyokabili uzalishaji wa ng'ombe wa nyama?

1 = Ukame (uhaba wa maji na malisho) [] 2 = Gharama kubwa za kununua dawa [] 3 = Ukosefu wa fedha [] 4 = Uwepo wa wanyama pori [] 5 = Wizi wa ng'ombe [] 6 = Kuenea kwa magonjwa [] 7 = Vifaa vichache vya kuogeshea ng'ombe katika maeneo mengine [] 10 = Ukosefu wa taasisi za mikopo []

16. Je! Shamba lako la malisho lina ukubwa gani kwa sasa ……………………… (Hekta)

17. Je! Unamiliki aina gani ya ng'ombe? Onyesha nambari katika kila kategoria?

kategoria	**Idadi**
Kigeni	
Mchanganiko wa kienyeji na kigeni	
Kienyeji	

18. Je! Muundo wako wa ng'ombe upoje? Jaza jedwali hapa chini

kategoria	**Idadi**		
	**Kienyeji**	**Kigeni**	**Mchanganiko wa kienyeji na kigeni**
Ng'ombe dume asie hasiwa			
Ngombe dume alie hasiwa/makisai			
Ngombe jike alie zaa			
Mitamba jike			

19. Ulipataje ng'ombe wako wa nyama? 1) Iliyorithiwa 2) ilinunuliwa katika soko la ndani 3) Zawadi 4) vingine (taja) …………

20. Chanzo chako kikuu cha mapato ni nini? ....

21. Je! Ni vyanzo vipi vingine vya mapato? 1) Uuzaji wa maziwa 2) Siagi / ghee 3) Ngozi 4) Uuzaji wa mazao 5) mengine (taja) ……………………………………

22. Je! Ni mapato kiasi gani kwa wastani kutoka vyanzo vingine kwa mwaka ...... Tz shillings

SEHEMU C: Uuzaji wa Ng'ombe (Uuzaji)

23. Je, umenunua ng'ombe wowote wa nyama tangu Januari mwaka jana? Ndio [ ]; Hapana [ ]; ikiwa ndio, ni wangapi ………

24. Je! Umeuza ng'ombe wowote wa nyama tangu Januari mwaka jana? Ndio [ ]; Hapana[ ]; ikiwa ndio, kamilisha jedwali hapo chini.

Kategoria	Idadi iliyo uzwa		Umri wa ng'ombe alieuzwa	Bei ya wastani	Sababu ya kuuza
	Kienyeji	Kigeni			
Ng'ombe dume asie hasiwa					
Ngombe dume aliehasiwa/makisai					
Ngombe jike alie zaa					
Mitamba jike					

Sababu za Kuuza: *1) Kulipa ada ya shule 2) Lipa bili za matibabu 3) Ununuzi wa chakula 4) Ng'ombe alikuwa anaumwa 5) Ununuzi wa vitu vya nyumbani 6) Mnyama alikuwa mzee (Kuondoa) 7) zingine (taja) ………*

25. Je! Kawaida huuza ng'ombe wako wapi? 1 = soko la minada ya awali [] 2 = Machinjio [] 3 = Wahudumu [] 4 = Wafanyabiashara [] 5.Bucha 6 = Soko la mnada wa upili. 7 = Zingine (taja) []

26. Kwa nini unapendelea soko hilo; 1 = matumizi ya vifaa vya kupimia [] 2 = Iko karibu na wafugaji 3 = Mazingira mazuri ya kuuza [] 4 = Hakuna soko lingine mbadala [] 6 = wengine… ..bainisha []

27. Je! Unapangaje bei gani za ng'ombe wa nyama?

1 = Chukua bei za soko [] 2 = hesabu ya gharama inayohusika [] 3 = nyingine (taja) …… []

28. Je! Unaamuaje bei kwa kila ng'ombe?

1) Uzito wa ng'ombe 2) Umri 3) Mwonekano wa mwili 4) vingine (taja) ……………

29. Je! Wewe ni wa vyama vya ushirika vya wakulima? Ndio [ ], Hapana [ ]; ikiwa ndio umefaidika na nini? 1) Mafunzo 2) Ziara ya kusoma 3) Uuzaji 4) Mengine (taja) ………………

30. Masoko ya ng'ombe yapo umbali kiasi gani kutoka nyumbani kwako? …… .. (kilometa).

31. Je! Unasafirishaje ng'ombe wako wa nyama kwenda sokoni ……………………………

32. Ni miezi ipi katika mwaka uliopita uliuza ng'ombe zaidi. Tafadhali kamilisha jedwali hapa chini.

Kategoria	Idadi ya Ng'ombe Zilizouzwa	Wastani wa Bei iliyouzwa
Januari		
Februari		
Machi		
Aprili		
Mei		
Juni		
Julai		
Agosti		
Septemba		
Oktoba		
Novemba		
Desemba		

33. Tafadhali onyesha gharama za uuzaji unazokabiliana nazo wakati wa kuuza ng'ombe wa nyama

**Gharama anuwai**	**Gharama ya jumla kwa ng'ombe wa nyama (Shilingi)**
Kazi (upakiaji na upakuaji ng'ombe)	
Gharama za usafirishaji	
Gharama iliyofichwa (wakati wa kusubiri, n.k.)	
Mawasiliano	
Ada ya soko	
Zingine (taja)	

34. Je! Unafahamu bei za ng'ombe wa nyama katika masoko mengine? Ndio [] Hapana [] ikiwa ndio, unapata wapi habari?

1) Wafugaji wenzangu 2) Mwanafamilia 3) NGOs

4) Chama cha Wakulima / vyama vya ushirika 5) Redio 6) kwenye magazeti

35. Je! Ni vizuizi vipi vikuu vya uuzaji wa ng'ombe wa nyama?1) Bei ya chini 2) Kukosa / idadi ndogo ya wafanyabiashara 3) Wafanyabiashara hushindwa kulipa 4) Kufikia soko la ngombe 5) Kutofikiwa kwa habari za soko 6) Hali duni ya barabara 7) Kujitenga 8) Vingine (taja)…………

36. Je! unanenepesha ng'ombe kabla ya kuuza? Ndio [ ]; Hapana [ ]; ikiwa ndio, ni nini chakula na gharama inayohusika kwa kila ng'ombe? .Kamilisha jedwali hapa chini.

**Gharama anuwai**	**Kiasi cha kitengo**	**Gharama ya kitengo**	**Kipindi cha kunenepesha**	**Umri wa ng'ombe wa kunenepesha**

37. Je! Bei ya wastani ya kuuza ya ng'ombe wa nyama alienenepeshwa ni ipi? ....Tshs.

ASANTE KWA TAARIFA YAKO YA THAMANI
