# Supplementary material for: Factors that influence market participation among traditional beef cattle farmers in the Meatu District of Simiyu Region, Tanzania
Source: PLoS One. 2021 Apr 1;16(4):e0248576. doi: 10.1371/journal.pone.0248576 (PMC8016299; doi:10.1371/journal.pone.0248576)
Supplement: S1 File — (ZIP) [file pone.0248576.s004.zip › S3A PDF. OLS Regression Output and Descriptive Statistics.pdf]

## S3A. OLS Regression Output and Descriptive Statistics

```
REGRESSION
  /MISSING LISTWISE
  /STATISTICS COEFF OUTS CI(95) R ANOVA
  /CRITERIA=PIN(.05) POUT(.10)
  /NOORIGIN
  /DEPENDENT NumberOfCattleSoldSinceJanuaryLastYear2019
  /METHOD=ENTER Age Educationlevel FamilyMembers CattleHerdSize
CattleFarmingExperience AccessToVeterinaryServices AccessToCredits
TotalLandSize LocalIndigenousBreeds BullsPerCattleHerd SteersPerCattleHerd
CowsPerCattleHerd HeifersPerCattleHerd AverageIncomeFromOtherSources
CattleFamersAssociationMembership DistanceFromHomeToCattleMarketsInKM
AccessToCattleMarketPriceInformation PracticingCattleFattening
AveragePriceOfCattleSoldSinceJanuaryLastYear2019.
```

# Regression

**Variables Entered/Removed[a]**

| Model | Variables Entered | Variables Removed | Method |
|---|---|---|---|

| | | | |
|---|---|---|---|
| 1 | AveragePriceOfferedPerBeefCattle, AccessToVeterinaryServices, NumberOfSteers_OxenPerHerdSize, CooperativesMembership, BeefCattleFarmingExperience, HouseholdSize, EducationLevelOfBeefCattleFarmer, AccessToCredits, AgeOfBeefCattleFarmer, NumberOfHeifersPerHerdSize, DistanceToBeefCattleMarket, AverageOff_FarmIncome, PracticingBeefCattleFattening, NumberOfBullsPerHerdSize, GrazingLandOwned, AccessToCattleMarketInformation, NumberOfCowsPerHerdSize, NumberOfLocalBreedsPerHerdSize, BeefCattleHerdSize[b] | . | Enter |

a. Dependent Variable: TotalNumberOfBeefCattleSoldToMarkets

b. All requested variables entered.

**Model Summary**

| Model | R | R Square | Adjusted R Square | Std. Error of the Estimate |
|---|---|---|---|---|
| 1 | .890[a] | .792 | .780 | 3.57872 |

a. Predictors: (Constant), AveragePriceOfferedPerBeefCattle, AccessToVeterinaryServices, NumberOfSteers_OxenPerHerdSize, CooperativesMembership, BeefCattleFarmingExperience, HouseholdSize, EducationLevelOfBeefCattleFarmer, AccessToCredits, AgeOfBeefCattleFarmer, NumberOfHeifersPerHerdSize, DistanceToBeefCattleMarket, AverageOff_FarmIncome, PracticingBeefCattleFattening, NumberOfBullsPerHerdSize, GrazingLandOwned, AccessToCattleMarketInformation, NumberOfCowsPerHerdSize, NumberOfLocalBreedsPerHerdSize, BeefCattleHerdSize

**ANOVA[a]**

| Model | | Sum of Squares | df | Mean Square | F | Sig. |
|---|---|---|---|---|---|---|
| 1 | Regression | 16283.515 | 19 | 857.027 | 66.917 | .000[b] |
| | Residual | 4277.618 | 334 | 12.807 | | |
| | Total | 20561.133 | 353 | | | |

a. Dependent Variable: TotalNumberOfBeefCattleSoldToMarkets

b. Predictors: (Constant), AveragePriceOfferedPerBeefCattle, AccessToVeterinaryServices, NumberOfSteers_OxenPerHerdSize, CooperativesMembership, BeefCattleFarmingExperience, HouseholdSize, EducationLevelOfBeefCattleFarmer, AccessToCredits, AgeOfBeefCattleFarmer, NumberOfHeifersPerHerdSize, DistanceToBeefCattleMarket, AverageOff_FarmIncome, PracticingBeefCattleFattening, NumberOfBullsPerHerdSize, GrazingLandOwned, AccessToCattleMarketInformation, NumberOfCowsPerHerdSize, NumberOfLocalBreedsPerHerdSize, BeefCattleHerdSize

**Coefficients[a]**

| Model | | Unstandardized Coefficients | | Standardized Coefficients | t | Sig. |
|---|---|---|---|---|---|---|
| | | B | Std. Error | Beta | | |
| 1 | (Constant) | -5.165 | 2.375 | | -2.175 | .030 |
| | AgeOfBeefCattleFarmer | .181 | .035 | .197 | 5.211 | .000 |

| | | | | | |
|---|---|---|---|---|---|
| EducationLevelOfBeefCattleFarmer | -.054 | .431 | -.003 | -.125 | .900 |
| HouseholdSize | -.046 | .060 | -.027 | -.758 | .449 |
| BeefCattleHerdSize | .639 | .114 | 2.703 | 5.602 | .000 |
| BeefCattleFarmingExperience | -.098 | .031 | -.127 | -3.170 | .002 |
| AccessToVeterinaryServices | -1.558 | 1.034 | -.095 | -1.506 | .133 |
| AccessToCredits | -1.750 | 1.254 | -.048 | -1.396 | .164 |
| GrazingLandOwned | .003 | .004 | .047 | .765 | .445 |
| NumberOfLocalBreedsPerHerdSize | .185 | .053 | .809 | 3.483 | .001 |
| NumberOfBullsPerHerdSize | .441 | .124 | .476 | 3.561 | .000 |
| NumberOfSteers_OxenPerHerdSize | .359 | .123 | .222 | 2.929 | .004 |
| NumberOfCowsPerHerdSize | .479 | .114 | 1.023 | 4.193 | .000 |
| NumberOfHeifersPerHerdSize | .520 | .111 | .512 | 4.673 | .000 |
| AverageOff_FarmIncome | 7.299E-7 | .000 | .260 | 4.590 | .000 |
| CooperativesMembership | -1.884 | .615 | -.088 | -3.063 | .002 |
| DistanceToBeefCattleMarket | .750 | .220 | .148 | 3.410 | .001 |
| AccessToCattleMarketInformation | -6.850 | .915 | -.432 | -7.487 | .000 |
| PracticingBeefCattleFattening | 12.097 | 1.590 | .399 | 7.609 | .000 |
| AveragePriceOfferedPerBeefCattle | 4.049E-6 | .000 | .110 | 3.152 | .002 |

**Coefficients[a]**

| Model | | 95.0% Confidence Interval for B | |
|---|---|---|---|
| | | Lower Bound | Upper Bound |
| 1 | (Constant) | -9.837 | -.493 |
| | AgeOfBeefCattleFarmer | .113 | .250 |
| | EducationLevelOfBeefCattleFarmer | -.901 | .793 |
| | HouseholdSize | -.164 | .073 |
| | BeefCattleHerdSize | .632 | .814 |
| | BeefCattleFarmingExperience | -.159 | -.037 |
| | AccessToVeterinaryServices | -3.593 | .477 |

| | | | |
|---|---|---|---|
| | AccessToCredits | -4.216 | .716 |
| | GrazingLandOwned | -.004 | .010 |
| | NumberOfLocalBreedsPerHerdSize | .081 | .290 |
| | NumberOfBullsPerHerdSize | .198 | .685 |
| | NumberOfSteers_OxenPerHerdSize | .118 | .600 |
| | NumberOfCowsPerHerdSize | .255 | .704 |
| | NumberOfHeifersPerHerdSize | .301 | .739 |
| | AverageOff_FarmIncome | .000 | .000 |
| | CooperativesMembership | -3.094 | -.674 |
| | DistanceToBeefCattleMarket | .317 | 1.182 |
| | AccessToCattleMarketInformation | -8.650 | -5.050 |
| | PracticingBeefCattleFattening | 8.969 | 15.224 |
| | AveragePriceOfferedPerBeefCattle | .000 | .000 |

a. Dependent Variable: TotalNumberOfBeefCattleSoldToMarkets

```
FREQUENCIES VARIABLES=Age FamilyMembers CattleFarmingExperience TotalLandSize
BullsPerCattleHerd SteersPerCattleHerd CowsPerCattleHerd HeifersPerCattleHerd
AverageIncomeFromOtherSources CattleHerdSize
NumberOfCattleSoldSinceJanuaryLastYear2019 DistanceFromHomeToCattleMarketsInKM
AverageSellingPriceOfFattenedCattle
AveragePriceOfCattleSoldSinceJanuaryLastYear2019
  /FORMAT=NOTABLE
  /STATISTICS=STDDEV MINIMUM MAXIMUM MEAN SUM
  /ORDER=ANALYSIS.
```

# Frequencies

**Statistics**

| | | AgeOfBeefCattleFarmer | HouseholdSize | BeefCattleFarmingExperience | GrazingLandOwned | NumberOfBullsPerHerdSize |
|---|---|---|---|---|---|---|
| N | Valid | 393 | 393 | 393 | 393 | 393 |
| | Missing | 0 | 0 | 0 | 0 | 0 |
| Mean | | 53.73 | 13.11 | 24.41 | 94.7557 | 11.4326 |
| Std. Deviation | | 8.623 | 4.431 | 9.814 | 124.21499 | 7.89641 |
| Minimum | | 35 | 6 | 10 | .00 | 1.00 |
| Maximum | | 75 | 25 | 50 | 802.00 | 40.00 |
| Sum | | 21114 | 5154 | 9595 | 37239.00 | 4493.00 |

**Statistics**

| | | NumberOfSteers_OxenPerHerdSize | NumberOfCowsPerHerdSize | NumberOfHeifersPerHerdSize | AverageOff_FarmIncome | BeefCattleHerdSize |
|---|---|---|---|---|---|---|
| N | Valid | 393 | 393 | 393 | 393 | 393 |
| | Missing | 0 | 0 | 0 | 0 | 0 |
| Mean | | 8.8931 | 23.9313 | 9.5929 | 2722646.3104 | 53.46 |
| Std. Deviation | | 4.79783 | 15.69684 | 7.31661 | 2618375.24002 | 30.895 |
| Minimum | | .00 | 4.00 | .00 | 200000.00 | 13 |
| Maximum | | 20.00 | 100.00 | 40.00 | 12000000.00 | 200 |
| Sum | | 3495.00 | 9405.00 | 3770.00 | 1070000000.00 | 21008 |

**Statistics**

| | | TotalNumberOfBeefCattleSoldToMarkets | DistanceToBeefCattleMarket | AverageSellingPriceOfFattenedCattle | AveragePriceOfferedPerBeefCattle |
|---|---|---|---|---|---|
| N | Valid | 393 | 393 | 24 | 354 |
| | Missing | 0 | 0 | 369 | 39 |
| Mean | | 5.0102 | 5.38 | 1066666.6667 | 415211.8644 |
| Std. Deviation | | 7.43131 | 1.617 | 96308.68247 | 207923.49262 |
| Minimum | | .00 | 1 | 1000000.00 | 150000.00 |
| Maximum | | 40.00 | 10 | 1200000.00 | 1100000.00 |
| Sum | | 1969.00 | 2113 | 25600000.00 | 146985000.00 |

```
FREQUENCIES VARIABLES=Sex MaritalStatus Educationlevel PrimaryOccupation
SecondaryOccupation AccessToVeterinaryServices MainSourceOfLabour
AccessToCredits CattleFamersAssociationMembership
CattlePriceFixingMethodWhenSelling WaysToDetermineThePriceOfCattleWhenBuying
WaysToTransportCattleToCattleMarket PlaceForSellingCattle
MarketPreferedForSellingCattle ReasonForSellingAtPreferedMarket
AccessToCattleMarketPriceInformation PracticingCattleFattening
  /ORDER=ANALYSIS.
```

## Frequencies

**Statistics**

| | | HouseHoldHead'sSex | MaritalStatusOfHouseHoldHead | EducationLevelOfBeefCattleFarmer | PrimaryOccupationOfHouseHoldhead | SecondaryOccupationOfHouseHoldHead |
|---|---|---|---|---|---|---|
| N | Valid | 393 | 393 | 393 | 393 | 393 |
| | Missing | 0 | 0 | 0 | 0 | 0 |

**Statistics**

| | | AccessToVeterinaryServices | MainSourceOfLabourForCattleFarmingInHousehold | AccessToCredits | CooperativesMembership | CattlePriceFixingMethodWhenSelling |
|---|---|---|---|---|---|---|
| N | Valid | 393 | 393 | 393 | 393 | 393 |
| | Missing | 0 | 0 | 0 | 0 | 0 |

**Statistics**

| | | WaysToDetermineThePriceOfCattleWhenBuying | WaysToTransportCattleToCattleMarket | PlaceForSellingCattle | MarketPreferedForSellingCattle | ReasonForSellingAtPreferedMarket |
|---|---|---|---|---|---|---|
| N | Valid | 393 | 393 | 393 | 393 | 393 |
| | Missing | 0 | 0 | 0 | 0 | 0 |

**Statistics**

| | | AccessToCattleMarketInformation | PracticingBeefCattleFattening |
|---|---|---|---|
| N | Valid | 393 | 393 |
| | Missing | 0 | 0 |

## Frequency Table

**HouseHoldHead'sSex**

| | | Frequency | Percent | Valid Percent | Cumulative Percent |
|---|---|---|---|---|---|
| Valid | Male | 393 | 100.0 | 100.0 | 100.0 |

**MaritalStatusOfHouseHoldHead**

| | | Frequency | Percent | Valid Percent | Cumulative Percent |
|---|---|---|---|---|---|
| Valid | Married | 377 | 95.9 | 95.9 | 95.9 |
| | Divorced | 8 | 2.0 | 2.0 | 98.0 |
| | Widowed | 8 | 2.0 | 2.0 | 100.0 |
| | Total | 393 | 100.0 | 100.0 | |

**EducationLevelOfBeefCattleFarmer**

| | Frequency | Percent | Valid Percent | Cumulative Percent |
|---|---|---|---|---|

| | | | | | |
|---|---|---|---|---|---|
| Valid | No formal education | 110 | 28.0 | 28.0 | 28.0 |
| | Primary education | 267 | 67.9 | 67.9 | 95.9 |
| | Secondary Education | 16 | 4.1 | 4.1 | 100.0 |
| | Total | 393 | 100.0 | 100.0 | |

**PrimaryOccupationOfHouseHoldhead**

| | | Frequency | Percent | Valid Percent | Cumulative Percent |
|---|---|---|---|---|---|
| Valid | Cattle farming | 393 | 100.0 | 100.0 | 100.0 |

**SecondaryOccupationOfHouseHoldHead**

| | | Frequency | Percent | Valid Percent | Cumulative Percent |
|---|---|---|---|---|---|
| Valid | Business | 8 | 2.0 | 2.0 | 2.0 |
| | Crop production | 385 | 98.0 | 98.0 | 100.0 |
| | Total | 393 | 100.0 | 100.0 | |

**AccessToVeterinaryServices**

| | | Frequency | Percent | Valid Percent | Cumulative Percent |
|---|---|---|---|---|---|
| Valid | No | 265 | 67.4 | 67.4 | 67.4 |
| | Yes | 128 | 32.6 | 32.6 | 100.0 |
| | Total | 393 | 100.0 | 100.0 | |

**MainSourceOfLabourForCattleFarmingInHousehold**

| | | Frequency | Percent | Valid Percent | Cumulative Percent |
|---|---|---|---|---|---|
| Valid | Family | 368 | 93.6 | 93.6 | 93.6 |
| | Both Farmily and hired | 25 | 6.4 | 6.4 | 100.0 |
| | Total | 393 | 100.0 | 100.0 | |

**AccessToCredits**

| | | Frequency | Percent | Valid Percent | Cumulative Percent |
|---|---|---|---|---|---|
| Valid | N0 | 375 | 95.4 | 95.4 | 95.4 |

| | | | | | |
|---|---|---|---|---|---|
| | Yes | 18 | 4.6 | 4.6 | 100.0 |
| | Total | 393 | 100.0 | 100.0 | |

**CooperativesMembership**

| | | Frequency | Percent | Valid Percent | Cumulative Percent |
|---|---|---|---|---|---|
| Valid | No | 335 | 85.2 | 85.2 | 85.2 |
| | Yes | 58 | 14.8 | 14.8 | 100.0 |
| | Total | 393 | 100.0 | 100.0 | |

**CattlePriceFixingMethodWhenSelling**

| | | Frequency | Percent | Valid Percent | Cumulative Percent |
|---|---|---|---|---|---|
| Valid | Take market prices | 393 | 100.0 | 100.0 | 100.0 |

**WaysToDetermineThePriceOfCattleWhenBuying**

| | | Frequency | Percent | Valid Percent | Cumulative Percent |
|---|---|---|---|---|---|
| Valid | Age | 7 | 1.8 | 1.8 | 1.8 |
| | Physical appearance | 386 | 98.2 | 98.2 | 100.0 |
| | Total | 393 | 100.0 | 100.0 | |

**WaysToTransportCattleToCattleMarket**

| | | Frequency | Percent | Valid Percent | Cumulative Percent |
|---|---|---|---|---|---|
| Valid | Trecking | 393 | 100.0 | 100.0 | 100.0 |

**PlaceForSellingCattle**

| | | Frequency | Percent | Valid Percent | Cumulative Percent |
|---|---|---|---|---|---|
| Valid | Auctions | 144 | 36.6 | 36.6 | 36.6 |
| | Middlemen | 249 | 63.4 | 63.4 | 100.0 |
| | Total | 393 | 100.0 | 100.0 | |

**MarketPreferedForSellingCattle**

| | | Frequency | Percent | Valid Percent | Cumulative Percent |
|---|---|---|---|---|---|
| Valid | Primary Market | 393 | 100.0 | 100.0 | 100.0 |

**ReasonForSellingAtPreferedMarket**

| | | Frequency | Percent | Valid Percent | Cumulative Percent |
|---|---|---|---|---|---|
| Valid | It is nearby the cattle farmers | 273 | 69.5 | 69.5 | 69.5 |
| | Good environment for selling | 120 | 30.5 | 30.5 | 100.0 |
| | Total | 393 | 100.0 | 100.0 | |

**AccessToCattleMarketInformation**

| | | Frequency | Percent | Valid Percent | Cumulative Percent |
|---|---|---|---|---|---|
| Valid | No | 152 | 38.7 | 38.7 | 38.7 |
| | Yes | 241 | 61.3 | 61.3 | 100.0 |
| | Total | 393 | 100.0 | 100.0 | |

**PracticingBeefCattleFattening**

| | | Frequency | Percent | Valid Percent | Cumulative Percent |
|---|---|---|---|---|---|
| Valid | No | 369 | 93.9 | 93.9 | 93.9 |
| | Yes | 24 | 6.1 | 6.1 | 100.0 |
| | Total | 393 | 100.0 | 100.0 | |

```
FILTER OFF.
USE ALL.
EXECUTE.
```

```
USE ALL.
COMPUTE filter_$=(SoldCattleSinceJanuaryLastYear2019 = 1).
VARIABLE LABELS filter_$ 'SoldCattleSinceJanuaryLastYear2019 = 1 (FILTER)'.
VALUE LABELS filter_$ 0 'Not Selected' 1 'Selected'.
FORMATS filter_$ (f1.0).
FILTER BY filter_$.
EXECUTE.
FREQUENCIES VARIABLES=Sex MaritalStatus Educationlevel PrimaryOccupation
SecondaryOccupation AccessToVeterinaryServices MainSourceOfLabour
AccessToCredits CattleFamersAssociationMembership
CattlePriceFixingMethodWhenSelling WaysToDetermineThePriceOfCattleWhenBuying
```

```
WaysToTransportCattleToCattleMarket PlaceForSellingCattle
MarketPreferedForSellingCattle ReasonForSellingAtPreferedMarket
AccessToCattleMarketPriceInformation PracticingCattleFattening
  /ORDER=ANALYSIS.
```

## Frequencies

**Statistics**

| | | HouseHoldHead'sSex | MaritalStatusOfHouseHoldHead | EducationLevelOfBeefCattleFarmer | PrimaryOccupationOfHouseHoldhead | SecondaryOccupationOfHouseHoldHead |
|---|---|---|---|---|---|---|
| N | Valid | 354 | 354 | 354 | 354 | 354 |
| | Missing | 0 | 0 | 0 | 0 | 0 |

**Statistics**

| | | AccessToVeterinaryServices | MainSourceOfLabourForCattleFarmingInHousehold | AccessToCredits | CooperativesMembership | CattlePriceFixingMethodWhenSelling |
|---|---|---|---|---|---|---|
| N | Valid | 354 | 354 | 354 | 354 | 354 |
| | Missing | 0 | 0 | 0 | 0 | 0 |

**Statistics**

| | | WaysToDetermineThePriceOfCattleWhenBuying | WaysToTransportCattleToCattleMarket | PlaceForSellingCattle | MarketPreferedForSellingCattle | ReasonForSellingAtPreferedMarket |
|---|---|---|---|---|---|---|
| N | Valid | 354 | 354 | 354 | 354 | 354 |
| | Missing | 0 | 0 | 0 | 0 | 0 |

**Statistics**

| | | AccessToCattleMarketInformation | PracticingBeefCattleFattening |
|---|---|---|---|
| N | Valid | 354 | 354 |
| | Missing | 0 | 0 |

## Frequency Table

**HouseHoldHead'sSex**

| | | Frequency | Percent | Valid Percent | Cumulative Percent |
|---|---|---|---|---|---|
| Valid | Male | 354 | 100.0 | 100.0 | 100.0 |

**MaritalStatusOfHouseHoldHead**

| | | Frequency | Percent | Valid Percent | Cumulative Percent |
|---|---|---|---|---|---|
| Valid | Married | 338 | 95.5 | 95.5 | 95.5 |
| | Divorced | 8 | 2.3 | 2.3 | 97.7 |
| | Widowed | 8 | 2.3 | 2.3 | 100.0 |
| | Total | 354 | 100.0 | 100.0 | |

**EducationLevelOfBeefCattleFarmer**

| | | Frequency | Percent | Valid Percent | Cumulative Percent |
|---|---|---|---|---|---|
| Valid | No formal education | 102 | 28.8 | 28.8 | 28.8 |
| | Primary education | 244 | 68.9 | 68.9 | 97.7 |
| | Secondary Education | 8 | 2.3 | 2.3 | 100.0 |
| | Total | 354 | 100.0 | 100.0 | |

**PrimaryOccupationOfHouseHoldhead**

| | | Frequency | Percent | Valid Percent | Cumulative Percent |
|---|---|---|---|---|---|
| Valid | Cattle farming | 354 | 100.0 | 100.0 | 100.0 |

**SecondaryOccupationOfHouseHoldHead**

| | | Frequency | Percent | Valid Percent | Cumulative Percent |
|---|---|---|---|---|---|
| Valid | Business | 8 | 2.3 | 2.3 | 2.3 |
| | Crop production | 346 | 97.7 | 97.7 | 100.0 |
| | Total | 354 | 100.0 | 100.0 | |

**AccessToVeterinaryServices**

| | | Frequency | Percent | Valid Percent | Cumulative Percent |
|---|---|---|---|---|---|
| Valid | No | 242 | 68.4 | 68.4 | 68.4 |
| | Yes | 112 | 31.6 | 31.6 | 100.0 |
| | Total | 354 | 100.0 | 100.0 | |

**MainSourceOfLabourForCattleFarmingInHousehold**

| | | Frequency | Percent | Valid Percent | Cumulative Percent |
|---|---|---|---|---|---|
| Valid | Family | 329 | 92.9 | 92.9 | 92.9 |
| | Both Farmily and hired | 25 | 7.1 | 7.1 | 100.0 |
| | Total | 354 | 100.0 | 100.0 | |

**AccessToCredits**

| | | Frequency | Percent | Valid Percent | Cumulative Percent |
|---|---|---|---|---|---|
| Valid | N0 | 338 | 95.5 | 95.5 | 95.5 |
| | Yes | 16 | 4.5 | 4.5 | 100.0 |
| | Total | 354 | 100.0 | 100.0 | |

**CooperativesMembership**

| | | Frequency | Percent | Valid Percent | Cumulative Percent |
|---|---|---|---|---|---|
| Valid | No | 301 | 85.0 | 85.0 | 85.0 |
| | Yes | 53 | 15.0 | 15.0 | 100.0 |
| | Total | 354 | 100.0 | 100.0 | |

**CattlePriceFixingMethodWhenSelling**

| | | Frequency | Percent | Valid Percent | Cumulative Percent |
|---|---|---|---|---|---|
| Valid | Take market prices | 354 | 100.0 | 100.0 | 100.0 |

**WaysToDetermineThePriceOfCattleWhenBuying**

| | | Frequency | Percent | Valid Percent | Cumulative Percent |
|---|---|---|---|---|---|
| Valid | Age | 7 | 2.0 | 2.0 | 2.0 |
| | Physical appearance | 347 | 98.0 | 98.0 | 100.0 |
| | Total | 354 | 100.0 | 100.0 | |

**WaysToTransportCattleToCattleMarket**

| | | Frequency | Percent | Valid Percent | Cumulative Percent |
|---|---|---|---|---|---|
| Valid | Trecking | 354 | 100.0 | 100.0 | 100.0 |

**PlaceForSellingCattle**

| | | Frequency | Percent | Valid Percent | Cumulative Percent |
|---|---|---|---|---|---|
| Valid | Auctions | 128 | 36.2 | 36.2 | 36.2 |
| | Middlemen | 226 | 63.8 | 63.8 | 100.0 |
| | Total | 354 | 100.0 | 100.0 | |

**MarketPreferedForSellingCattle**

| | | Frequency | Percent | Valid Percent | Cumulative Percent |
|---|---|---|---|---|---|
| Valid | Primary Market | 354 | 100.0 | 100.0 | 100.0 |

**ReasonForSellingAtPreferedMarket**

| | | Frequency | Percent | Valid Percent | Cumulative Percent |
|---|---|---|---|---|---|
| Valid | It is nearby the cattle farmers | 250 | 70.6 | 70.6 | 70.6 |
| | Good environment for selling | 104 | 29.4 | 29.4 | 100.0 |
| | Total | 354 | 100.0 | 100.0 | |

**AccessToCattleMarketInformation**

| | | Frequency | Percent | Valid Percent | Cumulative Percent |
|---|---|---|---|---|---|
| Valid | No | 128 | 36.2 | 36.2 | 36.2 |
| | Yes | 226 | 63.8 | 63.8 | 100.0 |
| | Total | 354 | 100.0 | 100.0 | |

**PracticingBeefCattleFattening**

| | | Frequency | Percent | Valid Percent | Cumulative Percent |
|---|---|---|---|---|---|
| Valid | No | 330 | 93.2 | 93.2 | 93.2 |
| | Yes | 24 | 6.8 | 6.8 | 100.0 |

| | | | | |
|---|---|---|---|---|
| Total | 354 | 100.0 | 100.0 | |

```
FREQUENCIES VARIABLES=Age CattleHerdSize FamilyMembers CattleFarmingExperience
AverageIncomeFromOtherSources TotalLandSize BullsPerCattleHerd
CowsPerCattleHerd HeifersPerCattleHerd SteersPerCattleHerd
  /FORMAT=NOTABLE
  /STATISTICS=STDDEV MAXIMUM MEAN
  /ORDER=ANALYSIS.
```

## Frequencies

**Statistics**

| | | AgeOfBeefCattleFarmer | BeefCattleHerdSize | HouseholdSize | BeefCattleFarmingExperience | AverageOff_FarmIncome |
|---|---|---|---|---|---|---|
| N | Valid | 354 | 354 | 354 | 354 | 354 |
| | Missing | 0 | 0 | 0 | 0 | 0 |
| Mean | | 54.52 | 54.46 | 13.41 | 25.04 | 2861864.4068 |
| Std. Deviation | | 8.288 | 32.290 | 4.433 | 9.867 | 2722123.80086 |
| Maximum | | 75 | 200 | 25 | 50 | 12000000.00 |

**Statistics**

| | | GrazingLandOwned | NumberOfBullsPerHerdSize | NumberOfCowsPerHerdSize | NumberOfHeifersPerHerdSize | NumberOfSteers_OxenPerHerdSize |
|---|---|---|---|---|---|---|
| N | Valid | 354 | 354 | 354 | 354 | 354 |
| | Missing | 0 | 0 | 0 | 0 | 0 |
| Mean | | 98.5565 | 11.7458 | 24.3249 | 10.0028 | 8.8277 |
| Std. Deviation | | 129.41010 | 8.22680 | 16.27957 | 7.50505 | 4.71346 |
| Maximum | | 802.00 | 40.00 | 100.00 | 40.00 | 20.00 |

```
USE ALL.
COMPUTE filter_$=(SoldCattleSinceJanuaryLastYear2019 = 0).
VARIABLE LABELS filter_$ 'SoldCattleSinceJanuaryLastYear2019 = 0 (FILTER)'.
VALUE LABELS filter_$ 0 'Not Selected' 1 'Selected'.
FORMATS filter_$ (f1.0).
FILTER BY filter_$.
EXECUTE.
FREQUENCIES VARIABLES=Age CattleHerdSize FamilyMembers CattleFarmingExperience
AverageIncomeFromOtherSources TotalLandSize BullsPerCattleHerd
CowsPerCattleHerd HeifersPerCattleHerd SteersPerCattleHerd
  /FORMAT=NOTABLE
  /STATISTICS=STDDEV MAXIMUM MEAN
  /ORDER=ANALYSIS.
```

## Frequencies

**Statistics**

| | | AgeOfBeefCattleFarmer | BeefCattleHerdSize | HouseholdSize | BeefCattleFarmingExperience | AverageOff_FarmIncome |
|---|---|---|---|---|---|---|
| N | Valid | 39 | 39 | 39 | 39 | 39 |
| | Missing | 0 | 0 | 0 | 0 | 0 |
| Mean | | 46.54 | 44.31 | 10.44 | 18.74 | 1458974.3590 |
| Std. Deviation | | 8.366 | 8.086 | 3.432 | 7.243 | 264294.44178 |
| Maximum | | 61 | 60 | 17 | 29 | 1800000.00 |

**Statistics**

| | | GrazingLandOwned | NumberOfBullsPerHerdSize | NumberOfCowsPerHerdSize | NumberOfHeifersPerHerdSize | NumberOfSteers_OxenPerHerdSize |
|---|---|---|---|---|---|---|
| N | Valid | 39 | 39 | 39 | 39 | 39 |
| | Missing | 0 | 0 | 0 | 0 | 0 |
| Mean | | 60.2564 | 8.5897 | 20.3590 | 5.8718 | 9.4872 |
| Std. Deviation | | 47.32236 | 2.30238 | 8.07689 | 3.63593 | 5.53872 |
| Maximum | | 150.00 | 12.00 | 35.00 | 11.00 | 20.00 |

```
FREQUENCIES VARIABLES=Sex Educationlevel MaritalStatus PrimaryOccupation
SecondaryOccupation MainSourceOfLabour AccessToVeterinaryServices
AccessToCredits CattlePriceFixingMethodWhenSelling
MarketPreferedForSellingCattle CattleFamersAssociationMembership
AccessToCattleMarketPriceInformation PracticingCattleFattening
  /ORDER=ANALYSIS.
```

# Frequencies

**Statistics**

| | | HouseHoldHead'sSex | EducationLevelOfBeefCattleFarmer | MaritalStatusOfHouseHoldHead | PrimaryOccupationOfHouseHoldhead | SecondaryOccupationOfHouseHoldHead |
|---|---|---|---|---|---|---|
| N | Valid | 39 | 39 | 39 | 39 | 39 |
| | Missing | 0 | 0 | 0 | 0 | 0 |

**Statistics**

| | | MainSourceOfLabourForCattleFarmingInHousehold | AccessToVeterinaryServices | AccessToCredits | CattlePriceFixingMethodWhenSelling | MarketPreferedForSellingCattle |
|---|---|---|---|---|---|---|
| N | Valid | 39 | 39 | 39 | 39 | 39 |

| | | | | | | |
|---|---|---|---|---|---|---|
| | Missing | 0 | 0 | 0 | 0 | 0 |

**Statistics**

| | | CooperativesMembership | AccessToCattleMarketInformation | PracticingBeefCattleFattening |
|---|---|---|---|---|
| N | Valid | 39 | 39 | 39 |
| | Missing | 0 | 0 | 0 |

# Frequency Table

**HouseHoldHead'sSex**

| | | Frequency | Percent | Valid Percent | Cumulative Percent |
|---|---|---|---|---|---|
| Valid | Male | 39 | 100.0 | 100.0 | 100.0 |

**EducationLevelOfBeefCattleFarmer**

| | | Frequency | Percent | Valid Percent | Cumulative Percent |
|---|---|---|---|---|---|
| Valid | No formal education | 8 | 20.5 | 20.5 | 20.5 |
| | Primary education | 23 | 59.0 | 59.0 | 79.5 |
| | Secondary Education | 8 | 20.5 | 20.5 | 100.0 |
| | Total | 39 | 100.0 | 100.0 | |

**MaritalStatusOfHouseHoldHead**

| | | Frequency | Percent | Valid Percent | Cumulative Percent |
|---|---|---|---|---|---|
| Valid | Married | 39 | 100.0 | 100.0 | 100.0 |

**PrimaryOccupationOfHouseHoldhead**

| | | Frequency | Percent | Valid Percent | Cumulative Percent |
|---|---|---|---|---|---|
| Valid | Cattle farming | 39 | 100.0 | 100.0 | 100.0 |

**SecondaryOccupationOfHouseHoldHead**

| | | Frequency | Percent | Valid Percent | Cumulative Percent |
|---|---|---|---|---|---|

| | | | | | |
|---|---|---|---|---|---|
| Valid | Crop production | 39 | 100.0 | 100.0 | 100.0 |

**MainSourceOfLabourForCattleFarmingInHousehold**

| | | Frequency | Percent | Valid Percent | Cumulative Percent |
|---|---|---|---|---|---|
| Valid | Family | 39 | 100.0 | 100.0 | 100.0 |

**AccessToVeterinaryServices**

| | | Frequency | Percent | Valid Percent | Cumulative Percent |
|---|---|---|---|---|---|
| Valid | No | 23 | 59.0 | 59.0 | 59.0 |
| | Yes | 16 | 41.0 | 41.0 | 100.0 |
| | Total | 39 | 100.0 | 100.0 | |

**AccessToCredits**

| | | Frequency | Percent | Valid Percent | Cumulative Percent |
|---|---|---|---|---|---|
| Valid | N0 | 37 | 94.9 | 94.9 | 94.9 |
| | Yes | 2 | 5.1 | 5.1 | 100.0 |
| | Total | 39 | 100.0 | 100.0 | |

**CattlePriceFixingMethodWhenSelling**

| | | Frequency | Percent | Valid Percent | Cumulative Percent |
|---|---|---|---|---|---|
| Valid | Take market prices | 39 | 100.0 | 100.0 | 100.0 |

**MarketPreferedForSellingCattle**

| | | Frequency | Percent | Valid Percent | Cumulative Percent |
|---|---|---|---|---|---|
| Valid | Primary Market | 39 | 100.0 | 100.0 | 100.0 |

**CooperativesMembership**

| | | Frequency | Percent | Valid Percent | Cumulative Percent |
|---|---|---|---|---|---|
| Valid | No | 34 | 87.2 | 87.2 | 87.2 |

| | | | | | |
|---|---|---|---|---|---|
| | Yes | 5 | 12.8 | 12.8 | 100.0 |
| | Total | 39 | 100.0 | 100.0 | |

**AccessToCattleMarketInformation**

| | | Frequency | Percent | Valid Percent | Cumulative Percent |
|---|---|---|---|---|---|
| Valid | No | 24 | 61.5 | 61.5 | 61.5 |
| | Yes | 15 | 38.5 | 38.5 | 100.0 |
| | Total | 39 | 100.0 | 100.0 | |

**PracticingBeefCattleFattening**

| | | Frequency | Percent | Valid Percent | Cumulative Percent |
|---|---|---|---|---|---|
| Valid | No | 39 | 100.0 | 100.0 | 100.0 |
