# Supplementary material for: Factors that influence market participation among traditional beef cattle farmers in the Meatu District of Simiyu Region, Tanzania
Source: PLoS One. 2021 Apr 1;16(4):e0248576. doi: 10.1371/journal.pone.0248576 (PMC8016299; doi:10.1371/journal.pone.0248576)
Supplement: Attachment — Submitted filename: PONE-D-20-32555_reviewer.pdf [file pone.0248576.s005.pdf]

PLOS ONE

Analysis of the Influencing Factors of Traditional Beef Cattle Farmers' Market Participation in the Meatu District of Simiyu Region, Tanzania.

--Manuscript Draft--

**Manuscript Number:**	PONE-D-20-32555
**Article Type:**	Research Article
**Full Title:**	Analysis of the Influencing Factors of Traditional Beef Cattle Farmers' Market Participation in the Meatu District of Simiyu Region, Tanzania.
**Short Title:**	Analysis of the Influencing Factors of Traditional Beef Cattle Farmers' Market Participation in Tanzania.
**Corresponding Author:**	Cornel Anyisile Kibona, MSc/Ph. D Student<br>Jilin Agricultural Uinversity<br>Jilin,Changchun, CHINA
**Keywords:**	Beef cattle; beef cattle breeds; beef meat; commercialization; traditional beef cattle farming systems; commercial beef cattle ranching systems; Ordinary least squares (OLS) model; Linear Regression; market participants; determinant factors; better income; food security; sustainable beef cattle supply; poverty reduction; Tanzania
**Abstract:**	Beef cattle farmers' market participation is a key pathway in assuring better income, food security, and a sustainable supply of beef cattle in the market, consequently poverty reduction. Nevertheless, the sector is well known for its low market participation. Therefore, this study examined the factors determining beef cattle farmers' market participation in the Meatu district of Simiyu region, Tanzania. The survey purposively and randomly selected a sample size of 393 beef cattle farmers. The cross-sectional data collected through interviews and questionnaires were analyzed using descriptive statistics and ordinary least squares (OLS) regression models. Descriptive statistics revealed that the average age(54.52), household size(13.41), farming experience(25.04), off-farm income(1,228$), grazing land owned(39.88ha), cattle herd size(54.46), cows(24.32), bulls(11.75), heifers(10), local breed cattle(53.42), and access to market information(63.8%) revealed privileged among market participants, hence influencing market participation. Furthermore, econometric inference revealed that for a beef cattle farmer to increase the volume of beef cattle sales in the market, the key determinants at P<0.05 included the price of beef cattle, farming experience, off-farm income, distance to market, age, cooperative membership, access to market information, cattle fattening practice, cattle herd size, cows, bulls, heifers, steers, and local breed cattle. An increasing linkage to cooperative marketing is recommended.
**Order of Authors:**	Cornel Anyisile Kibona, MSc
	Zhang Yuejie
**Additional Information:**	
**Question**	**Response**
**Financial Disclosure**<br><br>Enter a financial disclosure statement that describes the sources of funding for the work included in this submission. Review the submission guidelines for detailed requirements. View published research articles from *PLOS ONE* for specific examples.<br><br>This statement is required for submission and **will appear in the published article** if	The research is supported and funded by the National Beef-cattle Industrial Technology System and Industrial Economy Research Project under the Ministry of Agriculture in the People's Republic of China (PRC) (CARS-37).<br>The funders had no role in study design, data collection and analysis,decision to publish,or preparation of the manuscript.

Powered by Editorial Manager® and ProduXion Manager® from Aries Systems Corporation

the submission is accepted. Please make sure it is accurate.<br><br>**Unfunded studies**<br>Enter: *The author(s) received no specific funding for this work.*<br><br>**Funded studies**<br>Enter a statement with the following details:<br>• Initials of the authors who received each award<br>• Grant numbers awarded to each author<br>• The full name of each funder<br>• URL of each funder website<br>• Did the sponsors or funders play any role in the study design, data collection and analysis, decision to publish, or preparation of the manuscript?<br>• **NO** - Include this sentence at the end of your statement: *The funders had no role in study design, data collection and analysis, decision to publish, or preparation of the manuscript.*<br>• **YES** - Specify the role(s) played.<br><br>* typeset	
**Competing Interests**<br><br>Use the instructions below to enter a competing interest statement for this submission. On behalf of all authors, disclose any competing interests that could be perceived to bias this work—acknowledging all financial support and any other relevant financial or non-financial competing interests.<br><br>This statement **will appear in the published article** if the submission is accepted. Please make sure it is accurate. View published research articles from *PLOS ONE* for specific examples.	The authors have declared that no competing interests exist.

**NO authors have competing interests**<br><br>Enter: *The authors have declared that no competing interests exist.*<br><br>**Authors with competing interests**<br><br>Enter competing interest details beginning with this statement:<br><br>*I have read the journal's policy and the authors of this manuscript have the following competing interests: [insert competing interests here]*<br><br>* typeset	
**Ethics Statement**<br><br>Enter an ethics statement for this submission. This statement is required if the study involved:<br><br>• Human participants<br>• Human specimens or tissue<br>• Vertebrate animals or cephalopods<br>• Vertebrate embryos or tissues<br>• Field research<br><br>Write "N/A" if the submission does not require an ethics statement.<br><br>General guidance is provided below. Consult the submission guidelines for detailed instructions. **Make sure that all information entered here is included in the Methods section of the manuscript.**	The research work was first approved by the Jilin Agricultural University, the doctor of philosophy (PhD) proposal committee, China. The approved research work was then submitted to the Ministry of Livestock and Fisheries (MLF) of Tanzania for approval. The Ministry of Livestock and Fisheries (MLF) approved the study with reference number (AB.16/2020/01). During the data collection process, all ethical matters were dealt with accordingly; first, the participants' consent was obtained verbally, and then the detailed information in the questionnaire as consent was filled out by participants before answering the questions. The Ward Executive Officer (WEO) witnessed the consent.

Powered by Editorial Manager® and ProduXion Manager® from Aries Systems Corporation

**Format for specific study types**<br><br>**Human Subject Research (involving human participants and/or tissue)**<br>• Give the name of the institutional review board or ethics committee that approved the study<br>• Include the approval number and/or a statement indicating approval of this research<br>• Indicate the form of consent obtained (written/oral) or the reason that consent was not obtained (e.g. the data were analyzed anonymously)<br><br>**Animal Research (involving vertebrate animals, embryos or tissues)**<br>• Provide the name of the Institutional Animal Care and Use Committee (IACUC) or other relevant ethics board that reviewed the study protocol, and indicate whether they approved this research or granted a formal waiver of ethical approval<br>• Include an approval number if one was obtained<br>• If the study involved *non-human primates*, add *additional details* about animal welfare and steps taken to ameliorate suffering<br>• If anesthesia, euthanasia, or any kind of animal sacrifice is part of the study, include briefly which substances and/or methods were applied<br><br>**Field Research**<br><br>Include the following details if this study involves the collection of plant, animal, or other materials from a natural setting:<br>• Field permit number<br>• Name of the institution or relevant body that granted permission	
**Data Availability**<br><br>Authors are required to make all data underlying the findings described fully available, without restriction, and from the time of publication. PLOS allows rare exceptions to address legal and ethical concerns. See the PLOS Data Policy and FAQ for detailed information.	Yes - all data are fully available without restriction

A Data Availability Statement describing where the data can be found is required at submission. Your answers to this question constitute the Data Availability Statement and **will be published in the article**, if accepted.<br><br>**Important:** Stating 'data available on request from the author' is not sufficient. If your data are only available upon request, select 'No' for the first question and explain your exceptional situation in the text box.<br><br>Do the authors confirm that all data underlying the findings described in their manuscript are fully available without restriction?	
**Describe where the data may be found in full sentences. If you are copying our sample text, replace any instances of XXX with the appropriate details.**<br><br>• If the data are **held or will be held in a public repository**, include URLs, accession numbers or DOIs. If this information will only be available after acceptance, indicate this by ticking the box below. For example: *All XXX files are available from the XXX database (accession number(s) XXX, XXX.)*.<br>• If the data are all contained **within the manuscript and/or Supporting Information files**, enter the following: *All relevant data are within the manuscript and its Supporting Information files.*<br>• If neither of these applies but you are able to provide **details of access elsewhere**, with or without limitations, please do so. For example:<br><br>*Data cannot be shared publicly because of [XXX]. Data are available from the XXX Institutional Data Access / Ethics Committee (contact via XXX) for researchers who meet the criteria for access to confidential data.*<br><br>*The data underlying the results presented in the study are available from (include the name of the third party*	All relevant data are within the manuscript

and contact information or URL).<br>• This text is appropriate if the data are owned by a third party and authors do not have permission to share the data.<br><br>* typeset	
Additional data availability information:	

Manuscript
Click here to access/download;Manuscript;renamed_04ceb.docx

Analysis of the Influencing Factors of Traditional Beef Cattle Farmers' Market Participation in the Meatu District of Simiyu Region, Tanzania

Cornel Anyisile Kibona[1, 2, *], Zhang Yuejie[1]

[1]College of Economics and Management, Jilin Agricultural University, Changchun, Jilin, China.Zhang-yj@163.com

[2]Department of Agricultural Economics and Finance, Mwalimu Julius. K. Nyerere University of Agriculture and Technology, Musoma, Tanzania.

*Correspondence: cornelkibona@gmail.com.com (CAK)

Abstract

Beef cattle farmers' market participation is a key pathway in assuring better income, food security, and a sustainable supply of beef cattle in the market, consequently poverty reduction. Nevertheless, the sector is well known for its low market participation. Therefore, this study examined the factors determining beef cattle farmers' market participation in the Meatu district of Simiyu region, Tanzania. The survey purposively and randomly selected a sample size of 393 beef cattle farmers. The cross-sectional data collected through interviews and questionnaires were analyzed using descriptive statistics and ordinary least squares (OLS) regression models. Descriptive statistics revealed that the average age[illegible].52), household size(13.41), farming experience(25.04), off-farm income(1,228$), grazing land owned(39.88ha), cattle herd size(54.46), cows(24.32), bulls(11.75), heifers(10), local breed cattle(53.42), and access to market information(63.8%) revealed privileged among market participants, hence influencing market participatio[illegible]urthermore, econometric inference revealed that for a beef cattle farmer to

increase the volume of beef cattle sales in the market, the key determinants at *P<0.05* included the price of beef cattle, farming experience, off-farm income, distance to market, age, cooperative membership, access to market information, cattle fattening practice, cattle herd size, cows, bulls, heifers, steers, and local breed cattle. An increasing linkage to cooperative marketing is recommended.

Keywords: Beef cattle; Ordinary least squares (OLS) model; Linear regression; market participants; better income; food security ; sustainable beef cattle supply; poverty reduction; Tanzania.

1. Introduction

An increased volume of beef cattle sales through market participation has become an influential aspect in the commercialization of the traditional beef cattle sector in rural areas [20]. Globally, it is predicted that more than 600 million people keep beef cattle, of which 75% live in rural areas [7].

The beef cattle sector in Tanzania is one of the major agricultural production sectors which contributes to over 50% of the beef cattle farmer's household income, 5.9% to national GDP, employment, and export earnings [11-13,27,41]. These contributions are untapped the full potential due to the limited commercialization of the traditional beef cattle sector [21]. The limited commercialization (market participation) of the traditional beef cattle sector is due to numerous challenges faced by beef cattle farmers to access markets and prevents them from taking advantage of market opportunities [23].

Tanzania ranks third (3rd) as the leading producer of beef cattle in Africa and 11th in the world, with a beef cattle population reaching 34.5 million with a 2.8% annual growth rate [20,27,41]. Beef cattle farming in Tanzania is a habitual means of life in which 94% of total beef cattle herds are predominantly raised under the traditional beef cattle farming system (a free-range production system) and 6% raised under the commercial beef cattle ranching system [10,13]. The potential increase in beef cattle is high since Tanzania has vast and abundant natural resources for beef cattle; approximately 60 million hectares are rangelands suitable for grazing [11].

Given that 94% of beef cattle herds in Tanzania are raised under the traditional beef cattle farming system, supplying 95% of beef meat produced in the country, together with the high dependency of beef cattle farmers on income generated from the sale of beef cattle[11-13], government interventions have been undertaken to commercialize the traditional beef cattle sector through different policies, programs, trade, and market liberalization to make more effective contributions to the family food security and income of beef cattle farmers and the national economy [9,12]. Efforts undertaken to commercialize the traditional beef cattle sector include but not limited: encouraging beef cattle farmers with a large number of beef cattle to reduce their beef cattle herd and settle on land allocated for beef cattle grazing, linking beef cattle farmers to profitable markets, encouraging them to operate beef cattle fattening as a means to increasing the productivity of beef cattle and profitability, collecting and disseminating beef cattle market information to beef cattle farmers to increase the volume of beef cattle sales, enabling access to credit, input subsidizing, vaccination control and access to veterinary skills and services[9,15,18]. The expectations of traditional beef cattle commercialization have been regarded as the means of meeting the increasing demand for beef cattle and beef meat in

domestic and international markets and poverty reduction among beef cattle farmers [9,14]. Beef cattle commercialization typically leads to an increased diversity of marketed commodities and increased specialization, thereby triggering beef cattle farmers to increase their incomes by producing beef cattle, which gives the highest returns [19].

Despite the efforts of the Tanzania government to commercialize the traditional beef cattle sector[9,12], the efforts have not proved effective[illegible]aditional beef cattle farmers continue to live in poverty; the country has remained a net importer of quality beef meat(over 700Metric tons per year[illegible]despite that after Ethiopia and Sudan, Tanzania ranks third in Africa in terms of the number of beef cattle and 11th in the world; the existing beef meat processing abattoirs have been operating at less than 50% of their operational capacities[6,11-13,22]. This is an indication of a low supply of beef cattle in the market and poverty among beef cattle farmers. The traditional beef cattle sector in Tanzania still has tremendous untapped potential to increase income for poor rural beef cattle farmers and increase the supply of beef cattle to domestic and international markets if the sector is more commercialized by increasing the extent level of market participation (volume beef cattle sales). However, the traditional beef cattle sector is well known for its low market participation. The wealth protection and prestige accumulated by beef cattle exceed market incentives, thus jeopardizing the integration of beef cattle farmers into the organized market [28].

According to the Tanzania Livestock Master Plan (TLMP) [10], without the balance of beef cattle production, beef meat production, and sustainable supply of marketable surplus in the market, the projected beef meat production-consumption gap by 2031/32 is estimated to be 1.7million tons. The deficit in the projected beef meat demand is driven by an increasing human population, income growth, urbanization, tourists, and income elasticity of demand. This

indicates a potential opportunity to be utilized by beef cattle farmers through increasing marketable surplus to offer for sale in the market, thus improving their living standards.

It is estimated that due to low market participation among traditional beef cattle farmers in Tanzania, by the year 2021/22, the contribution of traditional beef cattle systems will decrease by 8.43%, while the commercial beef cattle ranching system will increase by 85.94% [10]. The commercial beef cattle ranching system is more market-oriented than the traditional beef cattle system. More efforts should be put to transform traditional beef cattle farmers from subsistence farming to market-oriented, since 95% of beef meat produced in Tanzania comes from the traditional beef cattle system. The potential of the traditional beef cattle system to contribute more to beef meat production is higher than commercial beef cattle ranching systems. It is estimated that by the year 2021/22, the total stocks under the traditional beef cattle system will be approximately 35 million beef cattle, while under commercial beef cattle ranching system will be only 1.3million beef cattle [10]. The untapped potential contribution of traditional beef cattle systems can be achieved by increasing the level of market participation among traditional beef cattle farmers.

The low market participation of beef cattle farmers leads to an insufficient supply of beef cattle for beef meat production. Additionally, low market participation leads to increased poverty among beef cattle farmers due to less cash generated from beef cattle sales. There should be some factors that sensitize beef cattle farmers to treat beef cattle farming as a business. These factors trigger beef cattle farmers to set aside a large volume of beef cattle for sale each year and then fatten them to produce beef cattle of good quality, which will earn high profits [6].

Therefore, the purpose of this study was to analyze the factors influencing the volume of beef cattle sales (market participation) among traditional beef cattle farmers. Understanding the

factors influencing beef cattle sales among beef cattle farmers is an essential foundation for establishing a sustainable development policy framework for maximizing rural economic growth and sustainable beef supply in the market [28].

2. Literature Review

2.1. Market Participation and its Role in Sustaining Beef Cattle Supply and Poverty Reduction

Market participation is related to commercialization, which refers to the gradual shift from traditional beef cattle farming to commercial beef cattle farming(market-oriented) or the act of farmers entering the beef cattle market and exchanging agricultural products for cash [5]. It is generally believed that the market participation of traditional beef cattle farmers is more important to the livelihoods of the rural population and its impact on the economic growth of developing countries [3]. It is also an important way for poor beef cattle farmers to secure better income and improve food security. Market participation is the first obstacle for beef cattle farmers to decide whether to participate in the beef cattle market or not. It is succeeded by deciding the quantity and quality of beef cattle sold in the market [28]. The more beef cattle supplied to the market, the higher the availability of beef cattle for beef meat production and export purposes. The improvement of market access and the existence of the market are crucial to traditional beef cattle farmers because they can attract the beef cattle sector and economic development [23]. Improving market access is essential to increase market participation and the volume of sales among beef cattle farmers [29]. It also stimulates infrastructure establishment and contributes to the overall development of rural areas and finally stimulates better living standards for beef cattle farmers [28]. Furthermore, market participation improves the welfare of beef cattle farmers by conducting market competition to reduce production costs and thereby

plunging food prices [17]. Higher productivity motivates beef cattle farmers to sell more of the marketable surplus for income generation. Generally, market participation ensures a continuous supply of beef cattle in the market and poverty reduction through income generated from the sale of beef cattle. Hence, analysis of factors affecting market participation (beef cattle sales) is fundamental to transforming traditional beef cattle production to market orientation, which then increases beef cattle supply and poverty reduction.

3. Materials and Methods

3.1. Description of the Study Site

The study was conducted in the Meatu District of the Simiyu Region. The region is located in northern Tanzania and southeast of Lake Victoria. The Simiyu Region, particularly the Meatu District, is dominated by traditional beef cattle farmers whose main economic activity is livestock keeping, contributing to over 80% of the district's economy and national beef cattle supply. The Simiyu Region is among the regions with a high population of beef cattle, with a significant contribution to the national beef cattle herds stock. It has an estimated total of 1,512,911 beef cattle herds [27], and covers an area of 25,212 square kilometers, with a population of 1,584,157 people [26-27]. The selected district has a population of 299,619 people [26-27]. The Meatu District receives an average rainfall between 600mm and 900mm per year and temperature ranges from 18^{0}C to 31^{0}C. Generally, grazing land occupies 95% of the total area and the remaining 5% is used for agricultural production. [26-27].

3.2. Ethics statement

The research work was first approved by the Jilin Agricultural University, the doctor of philosophy (PhD) proposal committee, China. The approved research work was then submitted to the Ministry of Livestock and Fisheries (MLF) of Tanzania for approval. The Ministry of Livestock and Fisheries (MLF) approved the study with reference number (AB.16/2020/01). During the data collection process, all ethical matters were dealt with accordingly; first, the participants' consent was obtained verbally, and then the detailed information in the questionnaire as consent was filled out by participants before answering the questions. The Ward Executive Officer (WEO) witnessed the consent.

3.3. Sampling Procedures

The study applied a multi-stage stratified sampling technique to select respondents of which are beef cattle farmers. The selection process of respondents at different stages involved both random and purposive sampling techniques. Stratified sampling is a sampling method that involves dividing the population into smaller groups called "strata." Stratified random sampling creates stratification based on members who share similar attributes [24]. Then, a random sample was selected from each stratum. In this study, the strata consisted of traditional beef cattle farmers. The Simiyu Region was selected as one of the major beef cattle producing regions. Thereafter, the major beef cattle producing district, namely, Meatu District, was selected and from the chosen district three villages were selected namely, nkoma, mwambegwa, and mwambiti. Beef cattle farmers were then purposely and randomly selected. The sample size of the respondents was 393 beef cattle farmers. Table 1 gives details of the population of respondents selected for each chosen study site.

Table 1. Sample size

Region	District	Villages	Sample population
Simiyu region	Meatu district	Nkoma	130
		Mwambiti	129
		Mwambegwa	133
Total			393

3.4. Data Collection

The cross-sectional primary data were collected using interviews aided by structured questionnaires and focused group discussions (FGD). The structured questionnaires captured three sections. Section I included the socioeconomic characteristics of beef cattle farmers, section II was designated for beef cattle production information, and section III for beef cattle sales (market participation) information.

3.5. Analytical model

This study applied both descriptive statistic and econometric models. Firstly, the socioeconomic characteristics of beef cattle farmers, beef cattle marketing activities, together with public services related factors were examined using descriptive statistics, which involved percentages, frequencies, means, and standard deviation.

Secondly, in econometric analysis, ordinary least squares (OLS) multiple linear regression model was applied to determine the factors influencing beef cattle farmers to increase the volume of beef cattle sales in the market. OLS is a mathematical modeling method that can be used to explain the relationship between a continuous dependent variable (beef cattle sold) and many independent variables [8]. The actual OLS model designated to analyze the factors influencing beef cattle sales of the sampled beef cattle farmers is as follows.

$$Y_i = \beta_0 + \beta_1 X_1 + \beta_2 X_2 + \beta_3 X_3 + \ldots\ldots\ldots\ldots + \beta_n X_n + \varepsilon_i \quad (1)$$

Where

Y_i denotes the number of beef cattle sold, β_0 is an intercept or constant, $\beta_{1,\ldots,..}$ β_n are the coefficients to be estimated, and $X_1,\ldots,$ and X_n represent the vectors of the explanatory variables and ε_i the error term.

The truncated OLS regression model for factors determining the volume of beef cattle sold was specified as

$$\begin{aligned} Number\ of\ beef\ cattle\ sold &= \beta_0 + \beta_1 Age + \beta_2 Education + \beta_3 Acces\ to\ market\ information \\ &+ \beta_4 Distance\ to\ market + \beta_5 Household\ size + \beta_6 Beef\ cattle\ herd\ size \\ &+ \beta_7 Farming\ experience + \beta_8 OffFarm\ income \\ &+ \beta_9 Access\ to\ veterinary\ services + \beta_{10} Access\ to\ credits \\ &+ \beta_{11} Grazing\ land\ owned + \beta_{12} Price\ of\ beef\ cattle \\ &+ \beta_{13} Beef\ cattle\ fattening\ practice + \beta_{14} Cooperative\ membership \\ &+ +\beta_{15} Cows + \beta_{16} Bulls + \beta_{17} Oxen + \beta_{18} Heifers + \beta_{19} Localbreeds + \varepsilon \end{aligned} \quad (2)$$

Table 2 below shows the hypothesized sign effects of the independent variables used in the OLS multiple regression model.

Table 2. Independent variables and their expected effects on the volume of beef cattle sold by beef cattle farmers.

Variables	Variable type	Measurements	Hypothesized signs
Age of a cattle farmers	Continuous	Age in years	±
Education level	Continuous	Years of schooling	+
Access to market information	Dummy	If 0=No,1=Yes	+
Distance from home to market	Continuous	Total kilometers	-
Household size	Continuous	Number of members	±
Beef cattle herd size	Continuous	Number of beef cattle	+
Experience in beef cattle farming	Continuous	In years	+
Off-farm income	Continuous	In Tanzanian shillings	±
Access to veterinary services	Dummy	If 0=No,1=Yes	+
Access to credits	Dummy	If 0=No,1=Yes	+
Grazing land owned	Continuous	In hectare	+
Price of beef cattle	Continuous	In Tanzanian shillings	+
Beef cattle fattening practice	Dummy	If 0=No,1=Yes	+
Cooperative membership	Dummy	If 0=No,1=Yes	+
Cows	Continuous	Number of cows	±
Bulls	Continuous	Number of bulls	+
Steers-Oxen	Continuous	Number of oxen	-

Heifers	Continuous	Number of heifers	±
Localbreeds	Continuous	Number of local breeds	-

3.6. Conceptual Framework

To commercialize the traditional beef cattle sector in Tanzania for a sustainable supply of beef cattle to the market and poverty reduction among beef cattle farmers, this study conceptualized that traditional beef cattle sector commercialization is dependent on the relationship between increased beef cattle sales (extent of market participation) and their determinants.

The relationship between the determinant (independent) variables and the dependent variable included in the study, together with the externalities, are illustrated in fig 1. Thus, an increased volume of beef cattle sales (dependent) was found to be influenced by age, education level, beef cattle herd size, farming experience, cooperative membership, grazing land owned, access to market information, access to credits, access to veterinary services, distance to market, price of beef cattle, off-farm income, household size, beef cattle fattening practice, number of cows owned, number of bulls owned, local breed owned, and crossbreeds owned. Based on the above variables, the volume of beef cattle sold (market participation) varies.

To justify the hypothesized variables, this study hypothesized that the age of beef cattle farmers has both positive and negative effects on the increased volume of beef cattle sales to the market because [32] found that there was a positive correlation between age and farmers' decision to increase the volume of beef cattle sales in the market, while Kgosikoma and Malope [31] found age to have a negative effect.

The study hypothesized that the education level of a beef cattle farmer has a positive impact on the increased volume of beef cattle sales in the market because farmers with higher education

level may have the ability to negotiate, obtain more market information, and adopt new technologies and innovations required to increase beef cattle productivity and beef cattle marketing skills [33].

A large beef cattle herd size means a more marketable surplus, which is an incentive to sell more beef cattle to the market [28], and the expansion of the beef cattle herd size stimulates vigorous market participation [34]. Hence, the study hypothesized that beef cattle herd size has a positive effect on the volume of beef cattle supplied to the market.

Furthermore, the study hypothesized that farming experience has a positive effect on the volume of beef cattle sold in the market because in unorganized marketing systems, particularly in rural areas, the effects of social networks accumulated over time can be captured through farming experience to enhance the search for potential customers [31].

Abafita et al. [35] in Ethiopia found that membership in cooperatives encourages beef cattle sales; therefore, this study expected cooperative membership to have a positive effect. Cooperatives help mobilize resources, share information, bargaining power, and promotion services [28].

Grazing land owned hypothesized a positive effect on beef cattle sales because sufficient grazing land encourages farmers to increase farm productivity and herd size, thus promoting beef cattle marketing due to the availability of marketable surplus.

The study hypothesized that obtaining accurate market information has a positive effect on beef cattle sales because beef cattle farmers need accurate market information to be able to correctly determine the number of beef cattle to be sold and the price to be obtained [23].

Access to farm credit was hypothesized to be positively related to beef cattle sales because credits increase access to investment resources and other services for production and beef cattle marketing strategies [28].

In this study, a positive relationship was hypothesized between access to veterinary services or extension services and the volume of beef cattle sold in the market because the beef cattle farmers exposed to veterinary services have a better perception of new technologies, such as artificial insemination (AI), a high-yielding beef cattle breed, disease control and treatment, and other modern farming methods, which promote the production of a marketable surplus, hence, improving livelihood [23].

Distance from home to the beef cattle market increases transaction costs, which hinders beef cattle farmers from participating in the market. Hence, this study assumed that the distance from home to the market is negatively related to the volume of beef cattle supplied to the market, [36] also revealed a negative relationship between market distance and beef cattle marketing activities.

Market prices for beef cattle are the ultimate motivation for beef cattle sellers [28]; hence, this study postulated that beef cattle prices have a positive effect on the volume of beef cattle sold in the market. A similar positive effect of the market price on market participation was found by Rutto et al. [34].

Studies by [16, 42] suggested that beef cattle farmers with high non-farm income are able to pay most of the bills without having to sell beef cattle, hence affecting the volume of beef cattle sold in the market, while [25] found that beef cattle farmers with high off-farm income offered more beef cattle for sale. Therefore, this study assumed that off-farm income has negative and positive effects on the volume of beef cattle sold in the market.

The larger the household size, the lower the market participation because big families have higher consumption demand, hence reducing marketable surplus to be offered for sale, contrary to contrast, larger households have the potential to engage more in beef cattle production and sales activities due to the availability of the labor force. In addition, large households need more income for school fees, medical care, and other livelihood needs, which put pressure on beef cattle herds to generate income [28]. Hence, this study hypothesized that household size has an indeterminate relationship with the volume of beef cattle sold to the market.

Beef cattle fattening improves beef cattle productivity, quality, and profitability of beef cattle. Farmers practicing fattening are more market-oriented; hence, the study postulated a positive relationship with the volume of beef cattle sold in the market.

Distinctive to this study, factors related to beef cattle, such as the number of bulls, cows, heifers, and steers-oxen owned together with beef cattle breeds (local and crossbreeds) kept were included to capture the replacement potential, beef cattle reproductive and availability of marketable surplus. Assumed that cows, heifers, and bulls have a positive impact on the volume of beef cattle sold in the market, cows and heifers increase the marketable surplus due to reproductive capacity, which motivates beef cattle farmers to offer more beef cattle for sale [28]. Generally, bulls are highly salable, which increases the volume of beef cattle sales in the market, while oxen availability lowers market participation because rural beef cattle farmers use oxen as the source of draught animal power; hence, a negative effect was hypothesized. Local breeds of beef cattle kept by traditional beef cattle farmers are characterized by slow-growing breeds with low market value; therefore, a negative influence on beef cattle sales was envisaged. Regarding crossbreed beef cattle, it was assumed that there was a positive effect on the volume of beef cattle sold in the market. Crossbreeds of beef cattle produce high-quality beef cattle, thus

increasing the value of beef cattle offered for sale [28]. In addition, crossbreeding is advantageous because of the breed that produces high-quality beef cattle through superior heterosis [37]. Hence, beef cattle flock characteristics are critical for marketing beef cattle surplus.

The framework also shows the interaction of some externalities supporting the increased volume of beef cattle sold (market participation). Based on the above factors, the expected outcomes are: poverty reduction, an increase in beef meat production, and an increase in the supply of beef cattle to the domestic and international markets, which finally leads to the growth of the national economy and foreign direct investment (FDI) in the beef cattle sector. The conceptual framework in figure 1 underpins the adoption and development of empirical analysis.

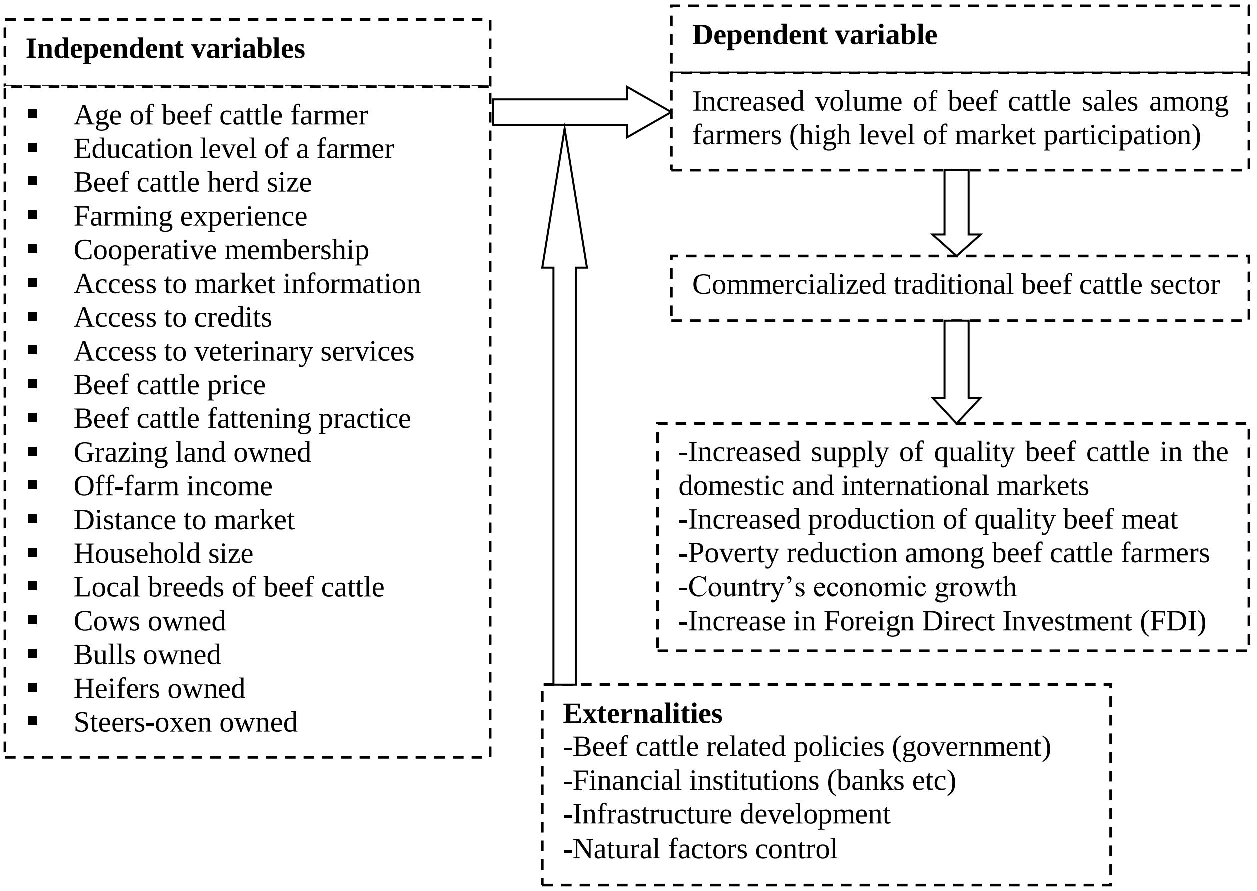

Figure 1.Concepture framework.

4. Results and Discussions

4.1. Descriptive analysis

4.1.1. Comparison of the Socioeconomic Characteristics of Market Participants and Non-Market Participants based on the Average Scores of Variables.

The differences in socioeconomic characteristics of non-market participants and market participants based on average scores of continuous variables are shown in Table 3. Out of 393 samples of beef cattle farmers indicated that 354(90.1%) beef cattle farmers had participated in the beef cattle market by selling an average of 5 beef cattle per year and only 39(9.9%) beef cattle farmers did not engage in beef cattle marketing. This indicates that the majority of beef cattle farmers depend on income generated from beef cattle sales to secure their financial situations. Among the market participants, the average age was 54.52 years, while non-market participants had an average age of 46.56 years. This indicates that both market participants and non-market participants were in the active age of the labor force, which is important in the adoption of new beef cattle production technologies and a willingness to engage in beef cattle marketing. The average household size among market participants was 13.41 higher than 10.44 of non-market participants, indicating a higher labor force potential for beef cattle production and marketing activities among market participants. It also shows that market participants had a higher beef cattle farming experience (25.04 years) than non-market participants (18.74 years). Beef cattle farming experience increases beef cattle productivity through the acquisition of skills and knowledge, thereby increasing beef cattle sales. It further captures the influence of social networks and links accumulated over time to enhance the search for potential customers [31, 28]. The market participants had an average of 54.46 beef cattle herd size, while non-market

participants had an average of 44.3 beef cattle herd size. This indicates that the beef cattle herd size of market participants is larger than the beef cattle herd size of non-market participants. The large herd size motivates beef cattle farmers to offer more for sale in the market due availability of marketable surplus. The results revealed that market participants had a large proportion of off-farm income (2,861,864.41Tanzanian shillings=1,228US$) per year than non-market participants (1,458,974 Tanzanian shillings=626 US$). Off-farm income increases farm productivity and marketable surplus if reinvested in beef cattle production, thereby increasing the chance of beef cattle farmers participating in the market. The mean of grazing land owned was higher among market participants, the mean of grazing land owned among market participants was 39.88 hectares and non-market participants was 24.38 hectares. Grazing land availability is important in beef cattle productivity, which enhances the availability of marketable surplus, which further increases the rate of market participation. The findings also show that the beef cattle flock characteristics such as cows, bulls, and heifers were higher on average among market participants than in non-market participants. The availability of these factors increases the availability of marketable surplus because they are concerned with beef cattle herd dynamics by increasing beef cattle productivity, hence promoting beef cattle sales among market participants. The steers-oxen average revealed higher among non-market participants. Steers-oxen are most used as a source of draught animal power in rural areas, which reduces marketable surplus to be offered for sale, thus, reduce the chances of participating in the market. The findings further discovered that neither non-market participants nor market participants raised crossbreeds. This shows that the traditional beef cattle sector is dominated by local breeds, which are characterized by slow growth and low productivity. This gives the potential to utilize the productivity vigor of crossbreeds to increase the marketable surplus. Beef cattle farmers raising hybrids are free to

benefit from the heterosis effect of producing high-quality beef cattle, which are of high market value [37]. Generally, the findings suggest that the higher means of variables among market participants positively influenced market participation and decision to offer more volume of beef cattle for sale in the market.

Table 3. Comparison of Socioeconomic characteristics of market participants and non-market participants based on the average scores of continuous variables

Variables	Overall(N=393)	Market participants(N=354)	Non-market participants(N=39)
Age	53.73	54.52	46.56
Household size	13.11	13.41	10.44
Beef cattle herd size	53.46	54.46	44.31
Farming experience	24.14	25.04	18.74
Off-farm income	2,722,646.31	2,861,864.41	1,458,974
Grazing land owned	38.35	39.88	24.38
Cows owned	23.93	24.32	20.36
Bulls owned	11.43	11.75	8.59
Steers-Oxen owned	8.89	8.83	9.49
Heifers owned	9.59	10.00	5.87
Local breeds owne[illegible]	52.52	53.42	44.31
Crossbreeds owned	0.0	0.00	0.00

4.1.2. Comparison of Socioeconomic Characteristics of Market Participants and Non-Market Participants based on Frequency and Percentage Scores of Categorical Variables.

Table 4 shows a comparison of descriptive percentages and frequencies of categorical variables between market participants and non-market participants. The findings revealed that among both market participants and non-market participants, 100% of males dominate beef cattle farming. This indicates a gender imbalance, female should be encouraged to engage in beef cattle farming since women are also responsible for farming, including beef cattle farming and food processing, as men migrate from rural areas to urban in search of employment [5]. Further analysis revealed that 28.8% of market participants had no formal education, while 20.5% of non-market participants had no formal education. This indicates the untapped potential of

education among market participants. A high level of education improves the ability to negotiate and access accurate market information. The findings show that 63.8% and 38.5% of market participants and non-market participants, respectively, had access to market information. This shows that compared with non-market participants, market participants were more exposed to market information. This means that obtaining accurate market information is an essential factor for beef cattle farmers to participate in the market and decision to increase the volume of beef cattle offered for sale. Given that farm credit is important for investing in beef cattle production and marketing activities, thus boosting beef cattle productivity, which increases the tendency to enter the market by increasing marketable surplus. [28].The findings show that out of the total sample (393), only 4.6% had access to farm credit. In addition, the results revealed that among market participants, only 4.5% of market participants and 5.1% of non-market participants had access to farm credit. This means that both market participants and non-market participants were less exposed to farm credit. Beef cattle farmers need a suitable and convenient arrangement as well as assistance in the establishment of a marketing system for securing farm credit. Regarding access to veterinary services, the results revealed that 31.6% and 41% of market participants and non-market participants, respectively, had access to veterinary services. The findings also revealed that among the market participants, 15% were engaged in cooperatives and 85% were not members of any cooperatives, while among non-market participants, 87.2% were not members of any cooperatives, but only 12.8% were engaged in cooperatives. This indicates that non-market participants were less involved in cooperative activities than market participants. An incentive should be set to attract beef cattle farmers to join cooperatives because cooperatives help mobilize resources, share information, bargaining power, and promotion services as well as cost reduction through economies of scale [28]. Furthermore, the results revealed that 6.8% of

market participants added value to their beef cattle before sale through beef cattle fattening, while none of the non-market participants practiced beef cattle fattening (value addition). This indicates that market participants are more market-oriented than non-market participants. In Tanzania, fattening beef cattle has been designated as one of several ways to increase beef cattle productivity through value addition [14]. Beef cattle of a Lower quality are fed with cotton husks, corn bran, or cottonseed cakes for three to four months to improve their quality by gaining weight, and then sell them at high prices [14]. Incentives to encourage beef cattle farmers to practice fattening are suggested.

Table 4. Comparison of socioeconomic characteristics between market participants and non-market participant based on frequency and percentage scores of categorical variables

Variables	Overall(N=393)		Market participants(N=354)		Non-market participants(N=39)	
	Frequency	Percentage	Frequency	Percentage	Frequency	Percentage
**Gender of a beef cattle farmer**						
Male	393	100	354	100.0	39	100.0
Female	0	0.0	0	0.0	0	0.0
**Education level**						
No education	110	28.0	102	28.8	8	20.5
Primary ed.	267	67.9	244	68.9	23	59.0
Secondary ed.	16	4.1	8	2.3	8	20.8
College ed.	0	0.0	0	0.0	0	0.0
**Access to market information**						
Yes	241	61.3	226	63.8	15	38.5
No	152	38.7	128	36.2	24	61.5
**Access to credits**						
Yes	18	4.6	16	4.5	2	5.1
No	375	95.4	338	95.5	37	94.9
**Access to veterinary services**						
Yes	128	32.6	112	31.6	16	41.0
No	265	67.4	242	68.4	23	59.0
**Membership to Cooperatives**						
Yes	58	14.8	53	15.0	5	12.8
No	335	85.2	301	85.0	34	87.2
**Practicing beef cattle fattening(value addition) before sale**						
Yes	24	6.1	24	6.8	0	0.0
No	369	93.9	330	93.2	39	100

4.2. Econometric analysis

4.2.1. Factors Influencing the Volume of Beef Cattle Sold in the Market by Beef Cattle Farmers

Table 5 shows the ordinary least squares (OLS) multiple linear regression outputs on the factors influencing the volume of beef cattle sold in the market by beef cattle farmers. The R^2 value from the OLS multiple regression analysis was 0.792. This shows that the independent variables account for approximately 79.2% of the change in the total volume of beef cattle sold in the market. The model was estimated using SPSS v22, and the model was a good fit and significant at $P<0.05$.

Holding other factors constant, OLS estimates indicated that the age of a beef cattle farmer, access to market information, distance to beef cattle market, beef cattle herd size, farming experience, off-farm income, price of beef cattle, beef cattle fattening practice, cooperative membership, and factors related to beef cattle, such as the number of bulls, cows, heifers, and steers-oxen owned together with the local breed of beef cattle kept were all statistically significant at a 5% significance level. While education level, household size, access to veterinary services, access to credits, and grazing land owned were all statistically insignificant at a 5% significance level.

The age of beef cattle farmers had a positive effect on the volume of beef cattle sold in the market and was statistically significant at the 5% significance level. The positive effect indicates that as the age of farmer increases, the volume of beef cattle sold in the market increases, age of a farmer is correlated with the decision on what volume of beef cattle to offer for sale. Age measures experience in beef cattle production and marketing. This result corresponds with the

study by Randela et al. [32], who found a positive association with the extent of market participation.

Considering that market information leads to improved beef cattle productivity, hence increased marketable surplus for sale in the market [23], However, access to market information negatively influenced the volume of beef cattle sold in the market and was statistically significant at the 5% significance level. This implies that access to inaccurate market information discourages beef cattle farmers from offering more beef cattle for sale.

The distance from home to the beef cattle market had a positive impact on the volume of beef cattle sold in the market and was statistically significant at the 5% significance level. According to [16], traveling for long distance to search for the beef cattle market consumes time and increases transportation costs, thus increasing marketing transaction costs, which hinder beef cattle farmers' market participation, however in rural areas, including the study area, the far located market offers a high and profitable price for beef cattle, hence motivating beef cattle farmers to offer more beef cattle for sale.

Beef cattle herd size had a positive effect on the volume of beef cattle sold in the market and was statistically significant at a 5% significance level. The results indicate that as beef cattle herd size increases, the volume of beef cattle offered for sale also increases. This study is in line with the studies by [31,28]. The beef cattle herd size is directly linked to the increase in the marketable surplus. This indicates that the larger the beef cattle herd size, the more likely it is to enter the market by selling the marketable surplus. [28].

Beef cattle farming experience indicated a significant negative effect on the volume of beef cattle sold in the market. This result does not correspond with the study by [1, 2, 23, 28, 38], which found a positive correlation between the increase in farming experience and market

participation rate. Knowledge and production techniques are acquired through farming experience and are known to be useful in production and marketing activities [38]; however, experienced beef cattle farmers in rural areas are inefficient in producing a marketable surplus, hence decreasing the volume of beef cattle to be offered for sale.

The off-farm income factor had a positive effect on the volume of beef cattle sold in the market and was statistically significant at a 5% significance level. This implies that keeping other factors constant, beef cattle farmers with a high off-farm income are likely to offer more volume of beef cattle for sale. Off-farm income is used as capital for beef cattle production and marketing related activities; hence, it increases the marketable surplus, which motivates beef cattle farmers to increase the volume of beef cattle to be sold in the market. This study corresponds with the studies by [25, 28], who reported that higher off-farm income motivates beef cattle farmers to participate more in the market by offering more beef cattle for sale, but this is not in line with the study by[39], who reported a negative correction between off-farm income and the decision to engage in beef cattle marketing

The price of beef cattle indicated a positive impact on the volume of beef cattle sold in the market by beef cattle farmers and it was statistically significant at a 5% significance level. This indicates that when the price of beef cattle goes up, beef cattle farmers increase the sales volume of beef cattle. Higher prices act as an incentive for beef cattle farmers to participate in the market by selling more marketable surplus.

The coefficient for practicing beef cattle fattening had a positive effect on the volume of beef cattle sold in the market and was statistically significant at the 5% significance level. Fattening practice adds value to beef cattle and, in turn, increases the productivity and market value, which

enhances the availability of marketable surplus, thereby increasing the volume of beef cattle sold in the market.

Furthermore, being a member of the cooperative was negatively associated with the volume of beef cattle sold in the market and was statistically significant at the 5% significance level. This indicates that cooperatives lack proper strategies to improve beef cattle farmer knowledge on beef cattle production and link farmers to social networks to get accurate market information about the beef cattle market.

Moreover, the addition of beef cattle flock characteristics on the determinants of the volume of beef cattle sold in the market is distinctive to this study. The coefficients of cows, heifers, bulls, and steers-oxen cattle had a positive effect on the volume of beef cattle sold in the market, with a significant level of 5%. Cows, heifers, and bulls are concerned in the multiplication of the beef cattle herd size through reproduction, hence increases the market surplus, which motivates beef cattle farmers to offer more for sale in the market. Steers-oxen are non-reproductive beef cattle; hence the availability of these stocks encourages beef cattle farmers to offer more beef cattle for sale. Additionally, the findings also showed that though local breeds of beef cattle are characterized by low growth and productivity compared to crossbreeds (hybrids), its coefficient positively correlated with the volume of beef cattle offered for sale in the market. This is because compared to crossbreeds, local breeds are more resistant to diseases, drought, and heat stress hence reduces the mortality rate which increases the marketable surplus to offer for sale in the market.

Table 5. Ordinary least squares (OLS) multiple linear regression estimates on the factors determining the volume of beef cattle sold in the market by beef cattle farmers N=393).

**Variables**	**Coefficients(β)**	**Std. Error**
Age of a cattle farmers	0.197*	0.035
Education level of beef cattle farmer	-0.003	0.431
Access to market information	-0.432*	0.915

Distance to market	0.148*	0.220
Household size	-0.027	0.060
Beef cattle herd size	2.703*	0.114
Farming Experience	-0.127*	0.031
Off-farm income	0.260*	0.000
Access to veterinary services	-0.095	1.034
Access to credits	-0.048	1.254
Grazing land owned	0.047	0.004
Price of beef cattle(market price)	0.110*	0.000
Practicing beef cattle fattening	0.399*	1.590
Cooperative membership	-0.088*	0.615
Cows owned	1.023*	0.114
Bulls owned	0.476*	0.124
Steers-oxen owned	0.222*	0.123
Heifers owned	0.512*	0.111
Local breeds owned	0.809*	0.053
Constant	-5.165	2.375
R Squared(R^2)	0.792(79.2%)	
Adjusted R squared(Adj.R^2)	0.780(78.0%)	

**Indicate significance level at 5 %($P<0.05$)*

5. Conclusio[illegible]

The market participation among poor beef farmers is an important way to ensure better income and improve food security to reduce poverty together with its impact on national economic growth. The study revealed that Out of 393 samples of beef cattle farmers indicated that 354(90.1%) beef cattle farmers had participated in the beef cattle market by selling an average of 5 beef cattle per year and only 39(9.9%) beef cattle farmers did not participate in the beef cattle market. Further analysis indicated that market participants were found to be influenced by socioeconomic characteristics such as age, farming experience, off-farm income, grazing land owned, and factors related to beef cattle such as beef cattle herd size, the number of heifers, bulls, and cows owned, which were higher on average among market participants than in non-market participants. In addition, the majority of market participants were found to have high access to market information than non-participants but highly dominated by less educated people

with less exposure to public services such as farm credits, veterinary services, and agricultural cooperative services. Education and public services should be improved among beef cattle farmers to sensitize their level of market participation for sustainable beef cattle supply in the market and poverty reduction among beef cattle farmers.

The econometric analysis further revealed that for the beef cattle farmers to increase the volume of beef cattle sales in the market, socioeconomic characteristics, beef cattle related factors, and public services are the key factors. Age of a farmer, farming experience, access to market information, distance to market, off-farm income, price of beef cattle, cooperative membership, beef cattle fattening practice, beef cattle herd size, number of cows, bulls, steers-oxen, and heifers owned together with local breed type kept were found to have a significant impact on the volume of beef cattle sold in the market. Since the sustainable supply of beef cattle to the market and poverty reduction are directly linked to beef cattle production, its productivity should be improved through the provision of veterinary services, farm credits, education, agricultural cooperative services, and new technologies such as encouraging farmers to practice beef cattle fattening and keeping crossbreeds as it was found that most of the beef cattle farmers kept local breeds. Keeping crossbreed increases productivity due to its productivity vigor hence increases the marketable surplus, therefore motivates beef cattle farmers to offer more beef cattle for sale in the market.

The policy recommendations for this study focus on agricultural and marketing cooperatives societies (AMCOS) in Tanzania, which deal with the production, processing, transporting, and marketing of various crops. AMCOS in Tanzania, which is under the Ministry of Agriculture, should expand the services to the livestock sector particularly the beef cattle farming sector to enhance beef cattle productivity and marketing. A strong and effective AMCOS dealing with the

beef cattle sector should be established to facilitate the access to farm inputs among the beef cattle farmers; find markets and supervise marketing of beef cattle; provide handling services at the auction markets while waiting for marketing; arrange for transportation of the beef cattle to the profitable markets; provide education and training on new technologies and entrepreneurial skills related to beef cattle marketing and production, and link beef cattle farmers to financial institutions to enhance the access to the credits. These strategies will sensitize beef cattle farmers to treat beef cattle farming as a business, hence increase market participation which further improves their livelihood and sustainable supply of beef cattle to the domestic and international markets. The success of agricultural and marketing cooperatives societies (AMCOS) in Tanzania will be determined by strong and committed leadership; committed participation; education; effective communication; equality; skilled leaders and managerial ability; financial stability; diversification, volume of business; and monitoring and evaluation together with adhering to accounting standards.

Author contribution

Author Zhang Yuejie conceptualized the study, funding acquisition, supervision, project administration, resources provision, and visualization. Author Cornel Anyisile Kibona managed the literature review, data curation, methodology, performed formal analysis, wrote the first draft, and prepared the final draft of the manuscript. Both authors read and approved the final manuscript.

Funding

The research is supported and funded by the National Beef Cattle Industrial Technology System and Industrial Economy Research Project under the Ministry of Agriculture in the People's Republic of China (PRC) (CARS-37).

Conflicts of Interest

The authors have declared that there is no any conflicts of interest exist.

References.

1. Agwu NM. Determinants of profitability among plantain marketers in Abia State, Nigeria. The Nigerian Journal of Development Studies.**2009**; 7(1): 49–58.

2. Agwu NM, Ibeabuchi JO. Socio-Economic Analysis of Wholesale Rice Marketers in Abia State, Nigeria. International Journal of SocialScience and Humanity.**2011**;1, (4): 285-288.

3. Barrett CB, Bellamere M, Osterloh S. "Household Level Livestock Marketing Behaviuor Among Northern Kenya and Southern Ethiopian Pastoralists." Cornell University Ithaca, New York ,USA,**2004**.

4. Challa M, Tilahun U. Determinants and Impacts of Modern Agricultural Technology Adoption in West Wollega : The Case of Gulliso District. J. Biol. Agric. Healthc.**2014**; 4, 63–78.

5. Esmael Y, Bekele A, Ketema M. Determinants of level of smallholder farmers participation in potato sales in Kofele District, Oromia Region, Ethiopia. J. Agric. Sci. Res. **2016;** 3, 23–30.

6. Kadigi RMJ, Kadigi IL, Laswai GH, Kashaigili JJ. The value chain of cattle and beef products in Ilemela and Magu Districts, Tanzania: Market access, opportunities and linkages. Academia Journal of Agricultural Research.**2013**; 1(8): 145-155.

7. Kruger B, Lammerts-Imbuwa L. Livestock Marketing in Namibia: Training Manual. Namibia National Farmers Union (NNFU), **2008**.

8. Maree JG. First steps in research. Van Schaik Publishers, Pretoria, **2007**.

9. MLD (Ministry of Livestock Development). National Livestock Policy (NLP), **2006**. Available on http://www.mifugo.go.tz.

10. MLF (Ministry of Livestock and Fisheries).Tanzania Livestock Master Plan, **2018**: Availableonhttps://www.mifugouvuvi.go.tz/uploads/projects/1553601793TANZANIA%20LIVESTOCK%20MASTER%20PLAN.pdf.

11. MLFD (Ministry of Livestock and Fisheries Development).Tanzania Livestock Modernization Initiative (TLMI). Government document, **2015.**Available on https://livestocklivelihoodsandhealth.org/wpcontent/uploads/2015/07/Tanzania_Livestock_Modernization_Initiative_July_2015.pdf.

12. MLFD (Ministry of Livestock and Fisheries Development).Livestock Sector Development Program. Government document, **2011.Available** on https://www.tanzania.go.tz/egov_uploads/documents/Livestock_Programe_sw.pdf.

13. MLFD (Ministry of Livestock and Fisheries Development).Tanzania Livestock Sector DevelopmentStrategy,**2010**.Availableonhttps://www.tanzania.go.tz/egov_uploads/documents/development_strategy-_Livestock_sw.pdf.

14. Mlote SN, Mdoe NSY, Isinika AC, Mtenga LA. Value Addition of Beef Cattle Fattening in the Lake Zone in Tanzania: Challenges and Opportunities. Livestock Research for Rural Development. **2012;** 24 (6) Article No 95

15. Mlote SN, Mdoe NSY, Isinika AC, Mtenga LA. Estimating Technical Efficiency of Small Scale Beef Cattle Fattening in the Lake Zone in Tanzania. Journal of Development and Agricultural Economics.**2013**; 5 (5): 197 – 207.

16. Negassa A, Jabbar M. Livestock Ownership, Commercial Off-take Rates and Their Determinants in Ethiopia. Research Report 9. International Livestock Research Institute (ILRI), Nairobi, Kenya, **2008**.

17. Omiti J, Otieno D, Nyanamba T, McCullough E. Factors influencing the intensity of market participation by smallholder farmers: A case study of rural and peri-urban areas of Kenya. Afr. J. Agric. Resour. Econ. **2009**;3, 57–82

18. Pica-Ciamarra UP, Baker D, Chassama J, Fadiga M, Nsiima L. Linking small holders to livestock markets: combining market and household survey data in Tanzania. A Paper Presented At the 4th Meeting about Statistics on Rural Development and Agriculture Households Income, from 9 – 11 November, 2011, Rio de Janeiro, Brazil. pp. 1 – 18.

19. Pingali, P. From subsistence to commercial production systems: The transformation of Asian agriculture. American Journal of agricultural Economics.**1997**; 79(2):628-634.

20. Rios AR, Shively, GE, Masters WA. Farm production and household market participation: Evidence from LSMS data. In Proceedings of the International Association of Agricultural Economist Conference,Beijing, China, 16–22 August **2009**.

21. Ruhangawebare GK. Factors affecting the level of commercialization among cattle keepers in the pastoral areas of Uganda. Thesis for Award for PhD degree at Makerere University, Kampala, Uganda, **2010**, 116pp.

22. **SAGCOT (**Southern Agriculture Growth Corridor).Opportunities for Investors in the Livestock Sector of Tanzania. SAGCOT Investment Partnership Program, Dar es Salaam, Tanzania.**2012**; 44pp

23. Sang HL, Nyein NK, Soojung A. Analysis of the Factors Influencing Market Participation among Smallholder Rice Farmers in Magway Region, Central Dry Zone of Myanmar. Sustainability. **2018**; 10, 4441; doi: 10.3390/su10124441.

24. Sharma G. Pros and cons of different sampling techniques. International Journal of Applied Research. **2017**; 3(7):749-52.

25. Stephen GM, Kimpei M, Mathew KK, Juma MM. The Factors That Influence Beef Cattle Marketing Efficiency and The Behavior Of Pastoralists: A Case Study In Kenya-Review. World Journal of Research and Review *(WJRR).***2018**; P51-58.

26. URT (United Republic of Tanzania). Population and Housing Census. Population Distribution by Administrative Areas. National Bureau of Statistics Ministry of Finance Dar es Salaam and Office of Chief Government Statistician President's Office, Finance, Economy and Development Planning, Zanzibar, Tanzania. **2012**; 264pp.

27. URT (United Republic of Tanzania).Economic Survey. Ministry of Finance and Planning Dodoma-Tanzania, **2018**.

28. Wen-Chi H, Sicelo ID. A Double Hurdle Estimation of Sales Decisions by Smallholder Beef Cattle Farmers in Eswatini.Sustainability. **2019**, 11, 5185; doi: 10.3390/su11195185

29. World Bank Agriculture for Development. World Development Report 2008; World Bank: Washington, DC, USA, **2008**.

30. Zezza A, Covarrubias K, Nsiima L. Livestock and livelihoods in rural Tanzania. A descriptive analysis of the 2009 National Panel Survey. Ministry Livestock and Fisheries Development, Dar es salaam, Tanzania. **2012**; 57pp

31. Kgosikoma K, Malope P. Determinants of market participation and the institutional constraints: Case study of Kweneng West, Botswana. J. Agric. Ext. Rural Dev. **2016**; 8, 178–186.

32. Randela R, Alemu ZG, Groenewald JA. Factors enhancing market participation by small-scale cotton farmers. Agrekon. **2008**; 47, 451–469.

33. Lubungu M, Chapoto A, Tembo G, Lusaka Z. Smallholder Farmers Participation in Livestock Markets: The Case of Zambian Farmers; Indaba Agricultural Policy Research Institute: Lusaka, Zambia, 2012.

34. Rutto JK, Odhiambo MO, Njuguna EM, Murithi FM. Determinants of market participation decisions in small ruminants' market by livestock keepers in Isiolo and Marsabit Districts, Kenya. Livest. Res. Rural Dev.**2013**; 25, 273–282.

35. Abafita J, Atkinson J, Kim CS. Smallholder commercialization in Ethiopia: Market orientation and participation. Int. Food Res. J. **2016**; 23, 1797–1807.

36. Corsi S, Marchisio LV, Orsi L. Connecting smallholder farmers to local markets: Drivers of collective action, land tenure and food security in East Chad. Land Use Policy **2017**; 68, 39–47.

37. Willy BT, Gemechu A. Determinants of market participation and financial profitability of smallholder dairy farming: The Case of Bako Tibe,West Showa, Ethiopia. Trends Agric. Econ. **2016**; 9, 29–44.

38. Kefyalew G. Analysis of Smallholder Farmer's Participation in Production and Marketing of Export Potential Crops: The Case of Sesame in Diga District, East Wollega Zone of Oromia Regional State. Master's Thesis,Addis Ababa University, Addis Ababa, Ethiopia, 2012.

39. Musah AB, Bonsu OAY, Seini W. Market participation of smallholder maize farmers in the upper west region of Ghana. Afr. J. Agric. Res. **2014**; 9, 2427–2435.

40. Zezza A, Covarrubias K, Nsiima, L. Livestock and livelihoods in rural Tanzania. A descriptive analysis of the 2009 National Panel Survey. Ministry Livestock and Fisheries Development, Dar es salaam, Tanzania.**2012**; 57pp

41. FAOSTAT (Food and Agriculture Organization of the United Nations Statistics). Regional FAO data groupings, **2019**.Retrievedon http://www.fao.org/faostat/en/#data/QL

42. Randolph TF, Schelling E, Grace D, Nicholson CF, Leroy JL, Cole DC, et al. Invited Review: Role of livestock in human nutrition and health for poverty reduction in developing countries. J. Anim. Sci.**2007**; 85, 2788–2800.
